# Standardized methodology for assessing the presence, variants and area of the interthalamic adhesion using anatomical MRI (SNAP-IA): multicentric validation on 565 healthy individuals and multiple neurological disorders

**DOI:** 10.1007/s00429-026-03097-6

**Published:** 2026-03-23

**Authors:** Julie P. Vidal, Gonzalo Forno, Michael Hornberger, Meritxell Bach Cuadra, Lola Danet, Vinod J. Kumar, Patrice Péran, Thomas Tourdias, Emmanuel J. Barbeau

**Affiliations:** 1 Centre de recherche Cerveau et Cognition (Cerco), UMR5549, CNRS – Université de Toulouse, Toulouse, France; 2 Univ Toulouse, Inserm, ToNIC, Toulouse, France; 3 Geroscience Center for Brain Health and Metabolism (GERO), Santiago, Chile; 4 Neuropsychology and Clinical Neuroscience Laboratory (LANNEC), Physiopathology Department – ICBM, Neuroscience and East Neuroscience Departments, Faculty of Medicine, University of Chile, Avenida Salvador 486, Providencia, Santiago, Chile; 5 Clinical Neurosciences, Clinical and Experimental Sciences, Faculty of Medicine, University of Southampton, Southampton, UK; 6 Center for Biomedical Imaging (CIBM), University of Lausanne, Lausanne, Switzerland; 7 Radiology Department (CHUV), Lausanne University Hospital, Lausanne, Switzerland; 8 Max Planck Institute for Biological Cybernetics, Tuebingen, Germany; 9 Neuroimagerie diagnostique et thérapeutique, CHU de Bordeaux, 33000 Bordeaux, France; 10 Univ. Bordeaux, INSERM, Neurocentre Magendie, U1215, 3300 Bordeaux, France

**Keywords:** Interthalamic adhesion, Thalamus, Standardized protocol, Stroke, Neurodevelopmental and neuropsychiatric disorders

## Abstract

The interthalamic adhesion (IA) connects both thalami. Emerging research suggests it may support thalamo-cortical connectivity and could be involved in neurodevelopmental and neuropsychiatric conditions. However, inconsistent MRI evaluation hinders progress on this subject. We developed SNAP-IA, a standardized anatomical imaging protocol for consistent IA identification and quantification. This work leveraged the expertise from seven research teams (Toulouse, Santiago, Southampton, Lausanne, Tübingen, and Bordeaux). SNAP-IA includes three steps: (1) determination of IA presence/absence on T1-weighted MRI; (2) classification of IA variants (simple, broad, double, bilobar, and filiform); (3) segmentation-based area assessment. It was tested on 500 controls (20–69 yo) and patients (stroke, schizophrenia, bipolar disorder, and ADHD) with 0.6–1 mm isotropic T1-weighted MRI (3T to 9.4T). SNAP-IA application achieved high inter-dataset agreement (mean Dice ≈ 0.92), with an average identification time of 35 s. The IA was absent in 22.8% of controls. Simple and broad variants constituted 95% of identified IA while some variants (double, filiform) were observed less frequently. At 3T, females had a higher presence rate (84.4%) than males (69.8%) and a larger IA area. ANCOVA indicated that both age and gender were highly predictive of IA area, decreasing by 0.25 mm²/year. At 9.4T, absence rates were significantly higher (34.6%) than at 3T (18.1%, *p* = 0.002). Mean IA area did not differ significantly between 3T and 9.4T. Patients with neurodevelopmental or neuropsychiatric disorders had two times less IA presence, with significantly smaller IA. SNAP-IA provides a reliable, reproducible framework for anatomical IA assessment across populations and MRI sequences, enabling future research into its structural and functional roles and supporting automated, large-scale AI studies.

**Supplementary Information:**

The online version contains supplementary material available at 10.1007/s00429-026-03097-6.

## Introduction

The Interthalamic Adhesion (IA) is an enigmatic midline structure that connects the medial borders of the two thalami through the third ventricle. Intriguingly, it is not universally present. Its absence has been reported in approximately 10% to 30% of individuals, depending on the population and methodology used (Nopoulos et al. 2001; Wong et al. 2021). It is also the only robust sexual brain dimorphism consistently reported in the literature, being more prevalent among women (Borghei et al. 2021; Ceyhan et al. 2008; Davie and Baldwin 1967; Nopoulos et al. 2001; Şahin et al. 2023; Vidal et al. 2024, 2025). When present, this structure displays different sizes and shapes (Borghei et al. 2020; Patra et al. 2022; Pavlović et al. 2020; Takahashi et al. 2008a, b, 2009; Tsutsumi et al. 2021). The correlation between increasing age and IA absence remains debated (Borghei et al. 2021; Şahin et al. 2023; Birnbaum et al. 2018; Takahashi et al. 2009; Sen 2005; Trzesniak et al. 2011).

Research suggests that the IA may be relevant for studying various neurodevelopmental and neuropsychiatric disorders potentially linked to abnormalities in midline brain structures (Andreasen et al. 1994; Shenton et al. 2001; Trzesniak et al. 2011; Takahashi et al. 2009). Additionally, IA study may serve as a valuable and accessible biomarker for detecting midline abnormalities in various pathologies, such as Alzheimer’s disease and multiple sclerosis (Forno et al. 2025; Fournet et al., submitted). It may also enhance our understanding of its role in cognition, particularly regarding interhemispheric communication (Damle et al. 2017; Borghei et al. 2020; Trzesniak et al. 2016; Vidal et al. 2024). However, the lack of a standardized methodology to evaluate IA on imaging has made it difficult to compare findings across studies (Snyder et al. 1998; Trzesniak et al. 2011).

The vast majority of MRI sequences used for IA identification are T1-weighted (T1w) (Borghei et al. 2020; Ceyhan et al. 2008; Damle et al. 2017; Kochanski et al. 2018; Landin-Romero et al. 2016; Meisenzahl et al. 2002; Nopoulos et al. 2001; Snyder et al. 1998; Takahashi et al. 2008a, b; Whitehead and Najim 2020). But the sequence parameters are highly variable, including slice number and thickness (1–5 mm), magnetic field strength (1–7T), and inter-slice gaps (0–5 mm) (Trzesniak et al. 2011). Thicker slices, poor resolution, and inter-slice gaps are all factors that can lead to errors in the detection of the IA, possibly resulting in higher reported absence rates in certain earlier studies (de Souza Crippa et al. 2006; Erbağcı et al. 2002; Snyder et al. 1998; Wong et al. 2021).

There is also considerable disparity in how the presence or absence of the IA is classified. Some studies have relied solely on coronal slices (de Souza Crippa et al. 2006; Erbağcı et al. 2002; Landin-Romero et al. 2016; Snyder et al. 1998), while others have used both coronal and axial slices (Kochanski et al. 2018; Meisenzahl et al. 2002; Nopoulos et al. 2001; Takahashi et al. 2008a, b; Trzesniak et al. 2016), coronal and sagittal slices (Ceyhan et al. 2008), or all three planes (Damle et al. 2017; Ettinger et al. 2007). Furthermore, there are inconsistencies in the slice threshold required for classifying the IA as present. Some studies required at least two coronal slices (de Souza Crippa et al. 2006; Erbağcı et al. 2002; Nopoulos et al. 2001; Snyder et al. 1998), while others used three contiguous coronal and axial slices (Takahashi et al. 2008a, b; Trzesniak et al. 2016). In contrast, several studies applied qualitative criteria (Ceyhan et al. 2008; Damle et al. 2017; Ettinger et al. 2007; Meisenzahl et al. 2002; Shimizu et al. 2008; Tsutsumi et al. 2021). One study used size as a criterion, requiring the IA to be greater than 1 mm for it to be considered present (Kochanski et al. 2018). Additionally, the presence of partial volume effects on MRI or “kissing thalami”, as described by Borghei et al. (2021) in individuals with narrow third ventricles, where the thalami appear joined on several slices, can significantly hinder IA identification.

A standardized approach is essential to improve consistency across studies, as illustrated in the field of automated segmentation, where multiple tools and methods are available for structures such as the hippocampus (Sánchez-Benavides et al. 2010; Fischl et al. 2012; Yushkevich et al. 2015; Thyreau et al. 2018; Sackl et al. 2024). Such standardization is also crucial for the accurate identification and harmonized characterization of the IA, particularly in clinical contexts. Building upon our previous work (Vidal et al. 2024), which itself integrated insights from Borghei et al. (2020) and Tsutsumi et al. (2021), and has already been adopted in recent studies (Vidal et al. 2025; Forno et al. 2025), our aim was to refine and expand a standardized IA analysis protocol. Specifically, we focused on enhancing the assessment of IA presence, anatomical variants, and area quantification using T1w MRI with standardized criteria and validated tools. This study integrated the effort of seven different teams and was tested across various datasets including healthy subjects covering a large age-span (20–69 yo), and patients with stroke, Alzheimer’s disease, neurodevelopmental or neuropsychiatric disorders, at various imaging resolutions (0.6–1 mm isotropic) and field strength (3T, 9.4T). This refined protocol aims to provide a robust, reproducible framework for IA characterization, supporting its use in both research and clinical settings. For the first time, we introduce a standardized protocol for measuring the IA area, offering a potentially more objective and practical alternative to variant classification for a wide range of research and clinical applications, including future artificial intelligence studies which require high quality annotated data.

##  Methods

###  Objectives

Our main objective was to develop and validate SNAP-IA, a standardized protocol derived from our previous work (Vidal et al. 2024). The present version represents a significant evolution, refined through dedicated meetings and extensive testing on multiple datasets across seven collaborating teams: Cerco & Tonic (Toulouse, France), GERO (Santiago, Chile), Clinical Neurosciences, Clinical and Experimental Sciences (Southampton, United Kingdom), CIBM (Lausanne, Switzerland), the Max Planck Institute (Tübingen, Germany), and the Neurocentre Magendie (Bordeaux, France). Key enhancements include the formalization of criteria for IA variant classification, the introduction of a novel “filiform” variant, and the implementation of a reproducible, segmentation-based area assessment with partial volume effect (PVE) correction.

We then aimed to (1) evaluate the learning curve and time efficiency required for raters to reliably apply SNAP‑IA; (2) validate the protocol by comparing IA prevalence and variant distribution in vivo against the most consistent findings in the literature in healthy subjects as an external reference; (3) compare IA metrics (presence, variants, area) across clinical groups (stroke, schizophrenia, bipolar disorder, ADHD) and healthy controls; (4) assess the impact of magnetic field strength (3T vs. 9.4T) on IA characterization.

###  Datasets used in the current study

**Fig. 1:**
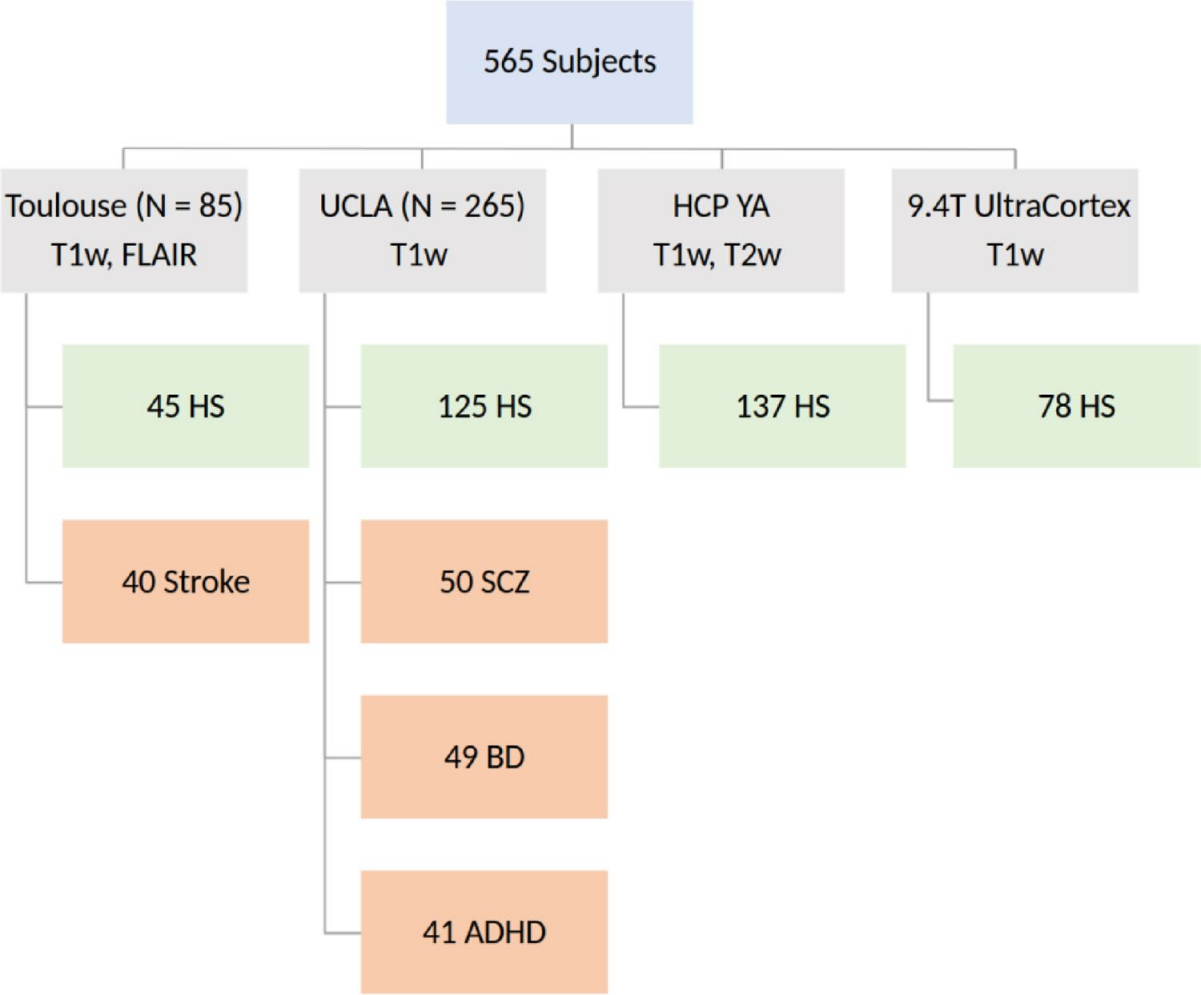
Diagram depicting all studies used to validate SNAP-IA including the number of individuals per study and experimental group. HS: healthy subjects, stroke: patients with isolated stroke of the thalamus, SCZ: schizophrenia, BD: bipolar disorders, ADHD: attention deficit hyperactivity disorders

565 subjects from 4 studies were characterized using the SNAP-IA protocol. Subjects came from different datasets detailed in Fig. 1.

- Toulouse MRI dataset

Patients with isolated stroke to the thalamus and matched healthy subjects were recruited in the Stroke Unit from the Toulouse University Hospital. For the first 20 patients and 20 healthy subjects, 3D T1-MPRAGE sequences were acquired on a Philips Achieva 3T scanner with the following parameters: 1 × 1 × 1 mm voxel size, TE = 3.7 ms, TR = 8.2 ms, flip angle = 8°, matrix = 240 × 240, spacing between slices = 0 mm. 3D T2-FLAIR (Fluid Attenuated Inversion Recovery) sequences parameters were: 1 × 1 × 1 mm voxel size, TE = 338 ms, TR = 8000 ms, TI = 2400 ms, matrix = 240 × 240, spacing between slices = 0 mm. For the next 20 patients and 25 healthy subjects, 3D T1-MPRAGE sequences were acquired on a Philips Achieva 3T scanner with the following parameters: 0.9 × 0.9 × 1 mm voxel size, TE = 8.1 ms, TR = 3.7 ms, flip angle = 8˚, matrix = 256 × 256, spacing between slices = 0 mm. 3D T2-FLAIR sequences parameters were: 1 × 1 × 1 mm voxel size, TE = 343 ms, TR = 8000 ms, TI = 2400 ms, matrix = 240 × 240, spacing between slices = 0 mm (Danet et al. 2015; 2017; Vidal et al. 2024).

b) UCLA MRI dataset

This dataset consists in publicly available MRI from patients with neurodevelopmental or neuropsychiatric disorders and healthy subjects from the Consortium for Neuropsychiatric Phenomics LA5c Study. 3D T1-MPRAGE sequences were acquired on a 3T Siemens Prisma scanner with the following parameters: 1 × 1 × 1 mm voxel size, TE = 2.26 ms, TR = 1.9 ms, flip angle = 7°, matrix = 256 × 256, spacing between slices = 0 mm (Gorgolewski et al. 2017).

c) HCP YA MRI dataset

The Human Connectome Project Young Adult (HCP YA) is a publicly available high-quality neuroimaging dataset from healthy young adults. MRI images were acquired using a Siemens 3T Connectome Skyra scanner. 3D T1-MPRAGE images were obtained using the following parameters: 0.7 × 0.7 × 0.7 mm voxel size, TE = 2.14 ms, TR = 2400 ms, TI = 1000 ms, flip angle = 8°, matrix = 224 × 224 mm, spacing between slices = 0 mm. The parameters for the 3D T2-SPACE sequences were: 0.7 × 0.7 × 0.7 mm voxel size, TE = 565 ms, TR = 3200 ms, matrix = 224 × 224 mm, spacing between slices = 0 mm (Van Essen et al. 2013).

d) 9.4T UltraCortex MRI dataset

9.4T UltraCortex is a publicly available ultra-high field MRI dataset from the UltraCortex repository. 3D T1-MPRAGE and MP2RAGE were acquired on a 9.4T Siemens Healthineers scanner with in total 11 different sequence parameters setups with resolutions of 0.6 to 0.8 mm (Mahler et al. 2024).

### Protocol

#### Protocol summary and availability

**Fig. 2:**
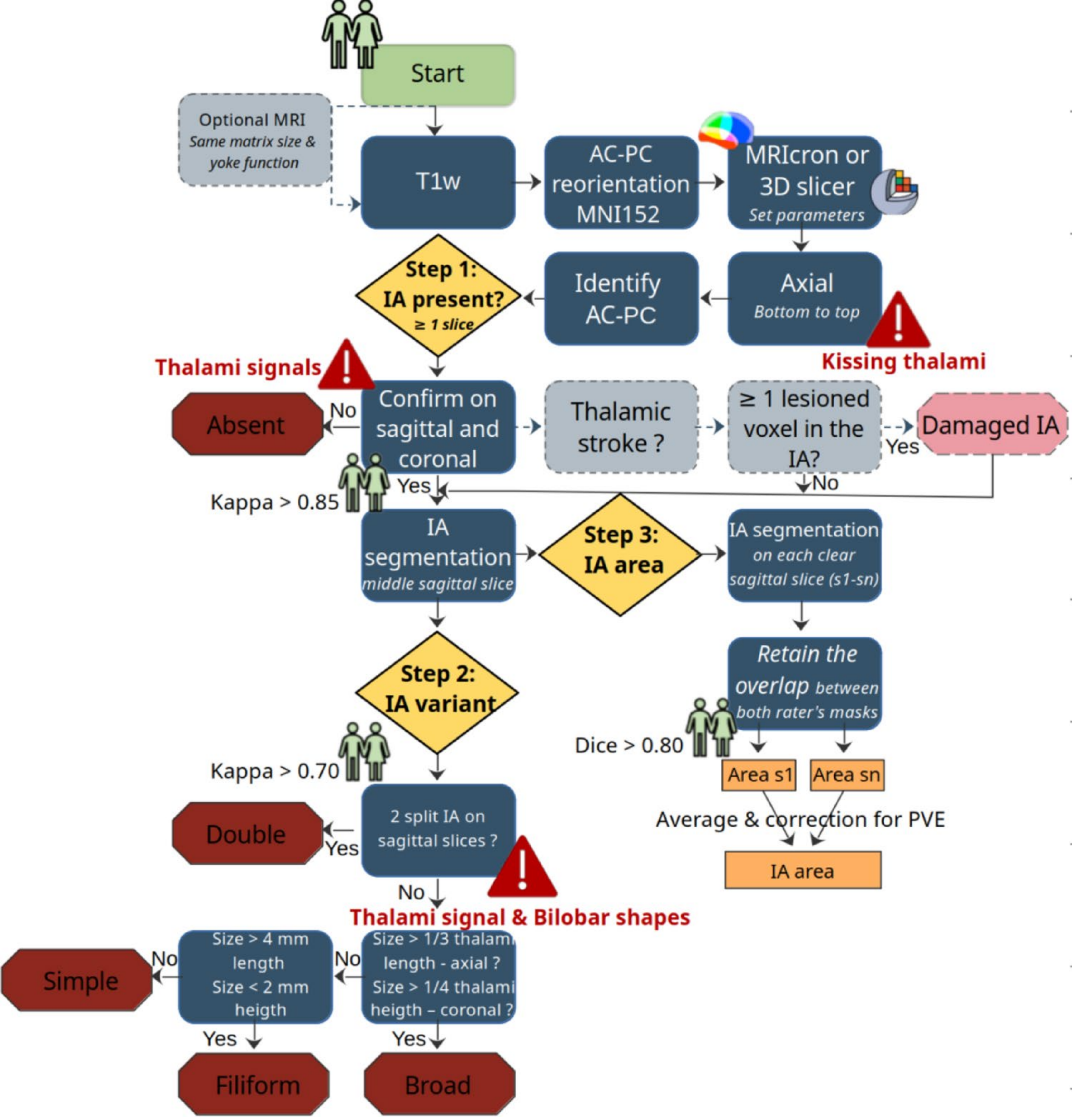
Schematic representation of SNAP-IA, illustrating the progression of the protocol from suggested parameters to specific assessment (highlighted in yellow diamonds). Step 1: presence or absence of the IA; Step 2: identification of IA anatomical variants; and Step 3: IA area

This protocol is called SNAP (Standardized Neuroanatomical Assessment Protocol) and is applied to the IA (SNAP-IA). A schematic summary of SNAP-IA is represented in Fig. 2. A detailed video protocol outlining the IA characterization process is publicly available (https://github.com/Julievidal8/SNAP-IA-protocol).

####  Protocol description

##### MRI requirements

To ensure accurate characterization of the IA, the MRI should have voxel size up to 1 mm³ isotropic with no inter-slice gap.

We recommend checking the quality of images, for example using MRIqc (Esteban et al. 2017), to ensure a good signal-to-noise ratio (SNR) for greater confidence in the results. This process also helps decrease bias-related noise by identifying subjects with a high Coefficient of Joint Variation (CJV), which could explain difficulties during assessments. Additionally, it is possible to identify artifacts and background noise using the Entropy Focus Criterion (EFC) and ensure spatial quality using Full Width at Half Maximum (FWHM).

All MRI sequences should be reoriented (e.g., MNI152 space as using FSL (fslreorient2std; Jenkinson et al. 2012) to facilitate the identification of the anterior and posterior commissures and prevent misidentification of the IA.

##### Raters and reproducibility

It is recommended that two independent raters assess independently the presence, anatomical variants, and any thalamic lesions extending into the IA. The use of MRIcron (Rorden 2025) or 3D Slicer (Pieper et al. 2004) is recommended, with a standardized zoom and contrast setting jointly defined by both raters and adjusted according to the MRI sequence type, to ensure consistency throughout the rating process. Dice between both raters can then be calculated. See below for expected Dice rates.

#### Step 1: IA presence or absence identification

The IA presence or absence is first assessed on axial slices from bottom to top. It is considered present if a structure connecting both thalami is visible in at least one slice between the anterior and posterior commissures. Its presence must then be confirmed in both coronal and sagittal views, with at least one plane validating previous observations.

##### Kissing thalami & tips

There may be occurrences of “kissing thalami” (Tsutsumi et al. 2021), where the thalami are fused at the expected IA location. PVE also occurs when a single voxel contains mixed tissue types, such as at the interface between the CSF-filled third ventricle and the thalamus. In such cases, direct signal intensity comparisons across different MRI sequences, as using MRIcron’s Yoke function, can provide valuable additional information.

##### Subject exclusion

In cases of kissing thalami, strong PVE or discordance between the two raters after attempts to reach a consensus, subject can be excluded from further analyses.

##### Pitfalls associated with sagittal slices and recommended guidelines

**Fig. 3:**
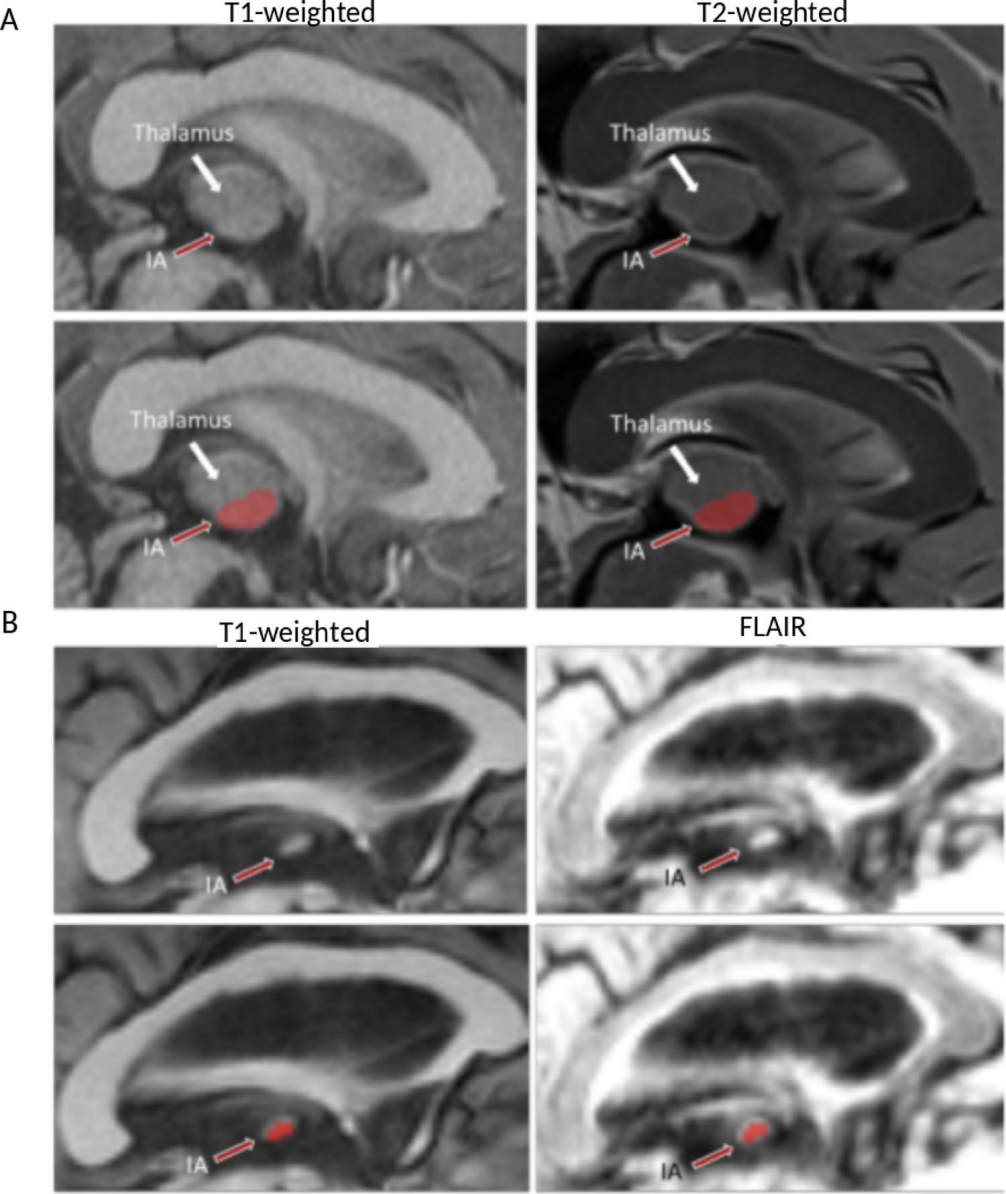
Sagittal slices from coregistered T1w and T2w or Flair MRI scans illustrating (A) A case from the HCP Young Adult database, where the IA signal overlaps with the thalamus. (B) A case from Toulouse database, where the IA is isolated from thalamic signal. The white arrow indicates the thalamus, while the red arrow highlights the IA. The top row displays the original MRI slices without segmentation, whereas the bottom row presents the corresponding slices with manual IA segmentation to help visualization

The IA may also appear as an isolated structure on sagittal slices when the thalami are sufficiently separated across the third ventricle (Fig. 3A). However, it often overlaps with thalamic signals, requiring careful differentiation (Fig. 3B). A useful tip for differentiating the thalamus from the IA is to navigate through sagittal slices starting from the one between both thalami. In these views, the thalamus exhibits notable size variation across slices, whereas the IA remains relatively consistent (see Supp. Figure 1).

##### Lesions in the IA

An IA is classified as damaged if at least one voxel of a lesion extends into it (Vidal et al. 2024).

##### IA’s presence/absence interrater agreement

To confirm interrater agreement and ensure reliable results, a kappa score must be calculated; if it is below 0.85, the raters must then discuss the discrepancies to reach a consensus. This threshold is based on our experience as it has been shown to be reachable with different groups of students working in pairs and aims to reflect a score of quality. Below, it indicates excessive variability in assessment, suggesting the need for additional rater training to improve consistency and reliability.

##### Optional additional MRI sequences to assess the presence or absence of the IA

**Fig. 4:**
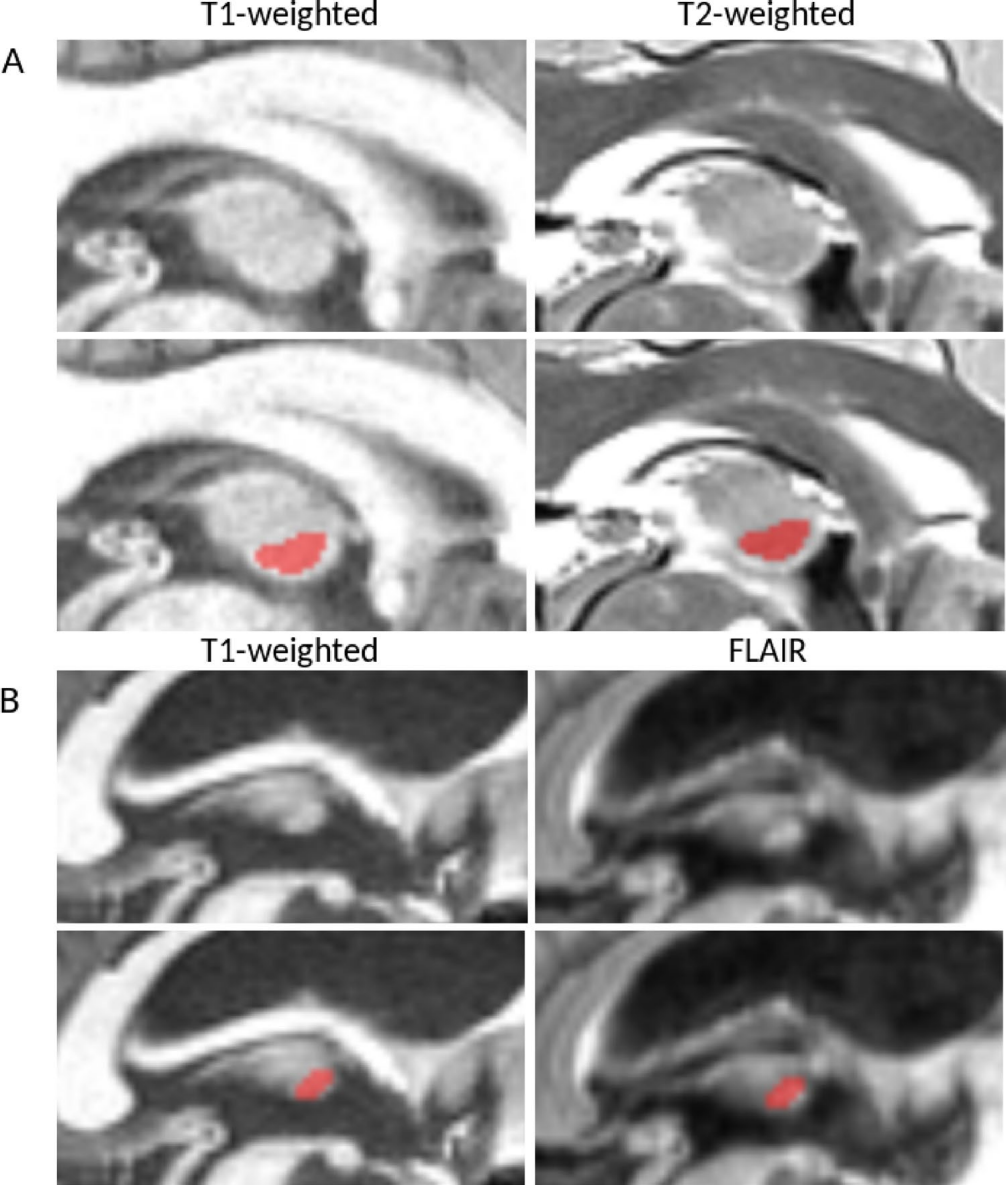
Contrast comparisons between sagittal slices of a T1w MRI and a coregistered sequence, including (A) T2w from the HCP Young Adult dataset (B) or FLAIR from the Toulouse dataset. The top row displays the original MRI slices without segmentation, while the bottom row presents the corresponding slices with manual IA segmentation to facilitate visualization

**Fig. 5:**
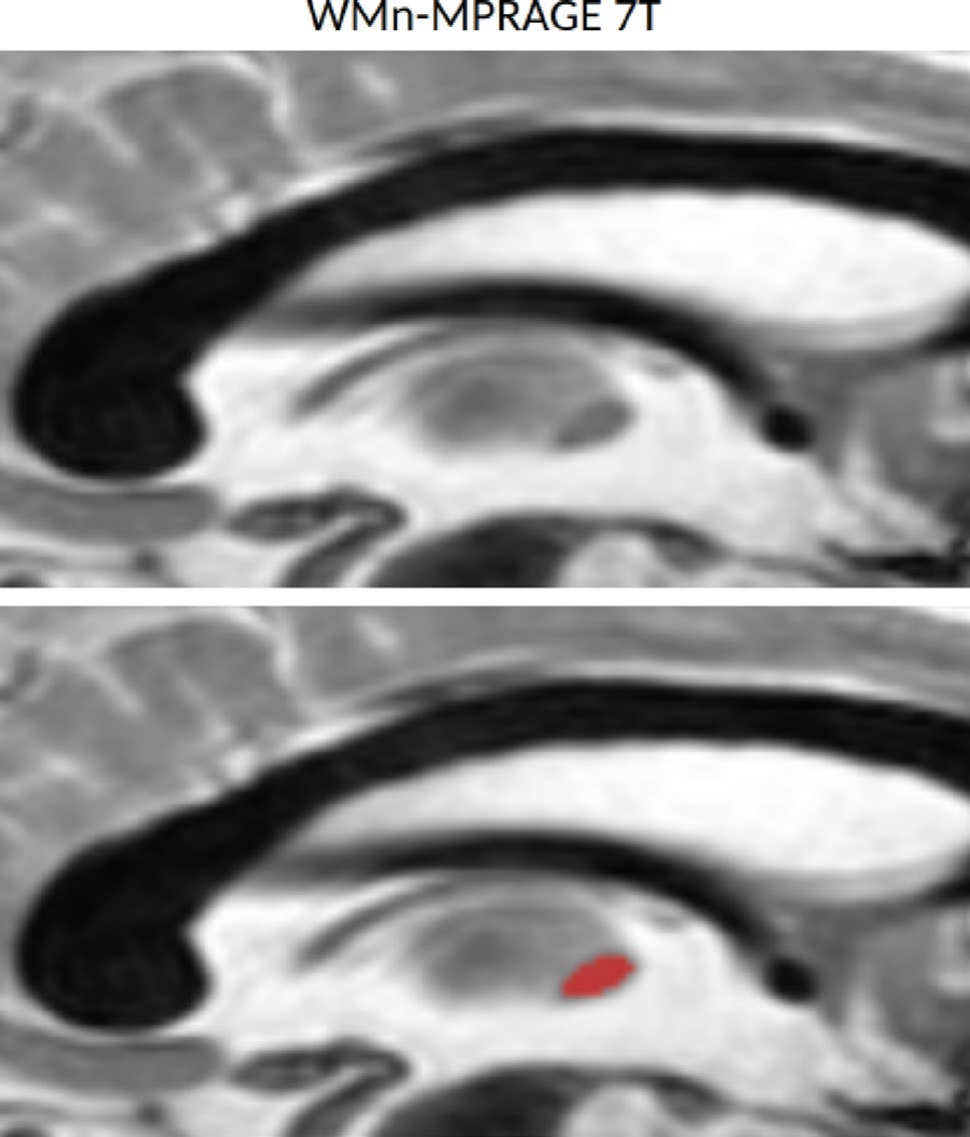
Illustration of a sagittal WMn-MPRAGE MRI slice acquired at 7T, based on the dataset reported by Su et al. (2019). The top image displays the original MRI slice without segmentation, whereas the bottom image presents the corresponding slice with manual IA segmentation to facilitate visualization

If available, optional MRI sequences can be used following the same logic as for T1w, for confirmation using a different contrast. The most relevant optional sequences include FLAIR, T2w (Fig. 4), and WMn images (Fig. 5). To ensure proper alignment, the optional sequence must be coregistered to T1w images (e.g., using FSL FLIRT with rigid transformation and 9 degrees of freedom, Jenkinson et al. 2012). To avoid the need for matrix reconstruction, which adjusts images to a common spatial resolution that can introduce artifacts and distortions (as shown for example in FLAIR’s Toulouse datasets, Fig. 3B), these sequences should share the same matrix size. Distortions may lead to misjudgments during assessment. In such cases, relying solely on T1w images may be recommended.

#### Step 2: IA’s anatomical variants

The classification of IA variants follows the framework described by Tsutsumi et al. (2021), with the exception of the tubular and rudimentary forms, which were not observed in our dataset, and the introduction of a new variant named “filiform”.

##### Simple and broad IA

**Fig. 6:**
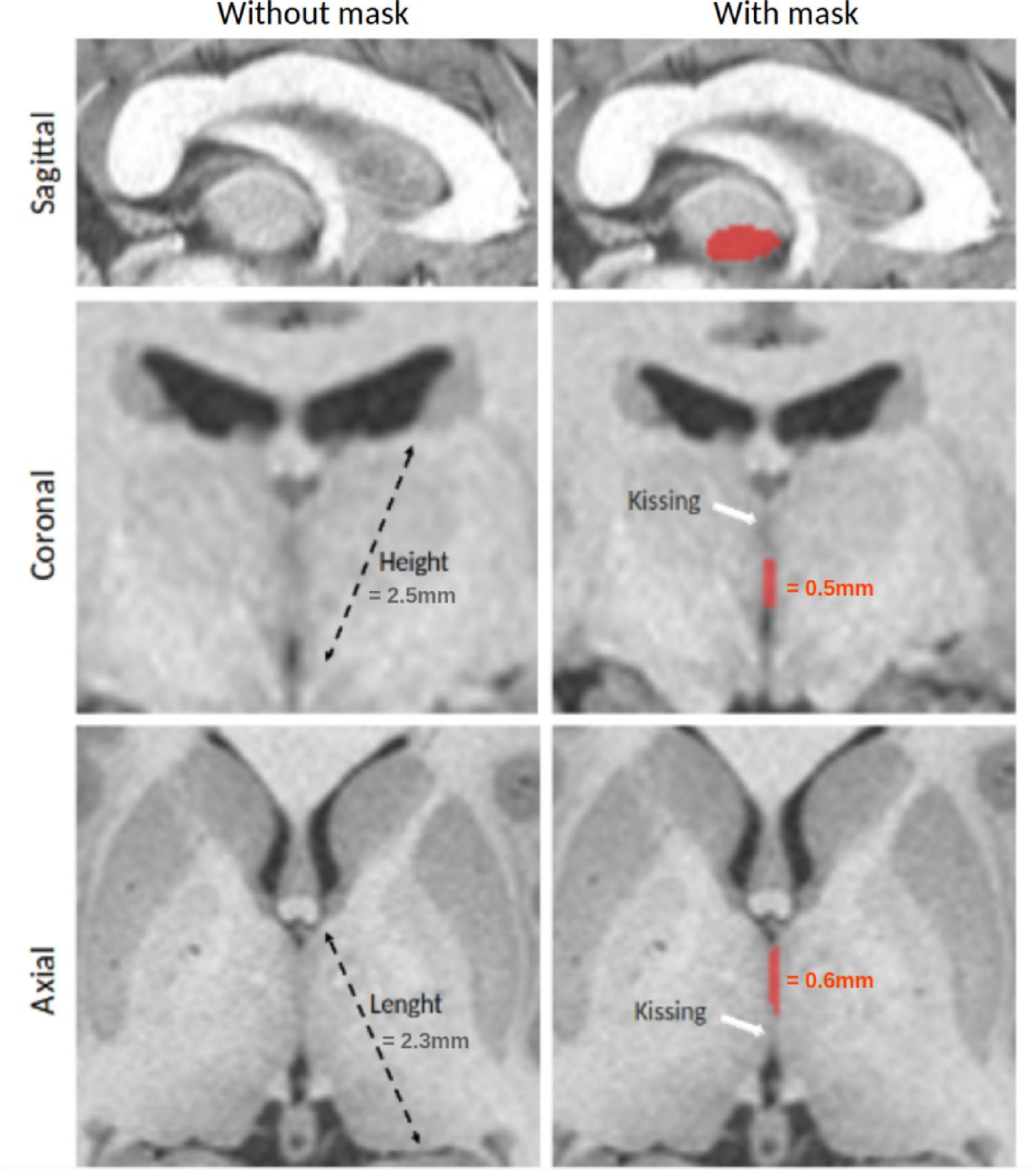
Illustration of a manually segmented sagittal slice of the IA on Toulouse dataset (Vidal et al. 2024). The IA can only be distinguished from the thalamic signal on the sagittal slice, providing crucial information for identifying regions where the signal does not originate from the IA on other slices. This distinction is essential for differentiating between the simple and broad IA forms. A broad IA is defined by two specific measurements relative to the thalamus. The height of the IA must be equal to or greater than 1/4 of the height of the thalamus, measured on the coronal slice that provides the largest view of the thalamus (greater than 2.5/4 = 0.625 in this example). The length of the IA must be equal to or greater than 1/3 (greater than 2.3/3 = 0.77 in this example) of the length of the thalamus, measured on the axial slice that provides the largest view of the thalamus. As the height of the IA is 0.5 < 0.625 and its length 0.6 < 0.77, this is a simple IA. To ensure accuracy, these measurements should be taken from the middle extremity of the thalamus and should consider its orientation. The segmentation mask generated on the sagittal slice facilitates precise height and length measurements, ensuring accurate classification and volumetric assessment. The left figures display the original MRI slices without segmentation’s mask, while the right figures present the corresponding slices with manual IA segmentation’s mask

The *simple* variant of the IA is characterized as a single, relatively small, round structure that connects both thalami. A *broad* IA is defined when its size reaches at least one-third of the thalamic length on axial slices or one-fourth of the thalamic height on the coronal slices that provide the largest view of the thalamus. To ensure precise measurements, dimensions should be taken from the left-middle to the right-middle for length, and from the upper-middle to the lower-middle part of the thalamus for height, taking into account its oval shape and orientation (Fig. 6). These relative measurements help account for individual variations in thalamic size. Measurements can be performed using a ruler on MRIcron or the virtual ruler feature in 3D Slicer. Given intra-individual variations in thalamic size and the fact that the left thalamus is reported as larger than the right in some studies (Burggraaff et al. 2021), we recommend comparing IA size to the same thalamic side chosen by the rater.

##### Sagittal slice segmentation tips

To differentiate the IA from thalami signals and to distinguish the simple variant from the broad variant, we recommend segmenting the IA on the sagittal slice where it is clearly distinguishable from the thalamus. Raters must however avoid classifying the IA variant based solely on sagittal slices, as thalamic edges may appear sharper, potentially leading to the misidentification of a broad variant. This approach provides a well-defined visual reference, which can then be used as a guiding mask for measurements on axial and coronal slices, ensuring greater accuracy in classification (Fig. 6). Standardized zoom and contrast should be maintained between raters for segmentation; however, it may differ from the previously used settings to allow for a more focused view.

##### Double variant and bilobar shape

The *double variant* is characterized by the presence of two separate IAs confirmed in each MRI plane. Notably, some studies have reported more than two IAs per subject (Tsutsumi et al. 2021).

**Fig. 7:**
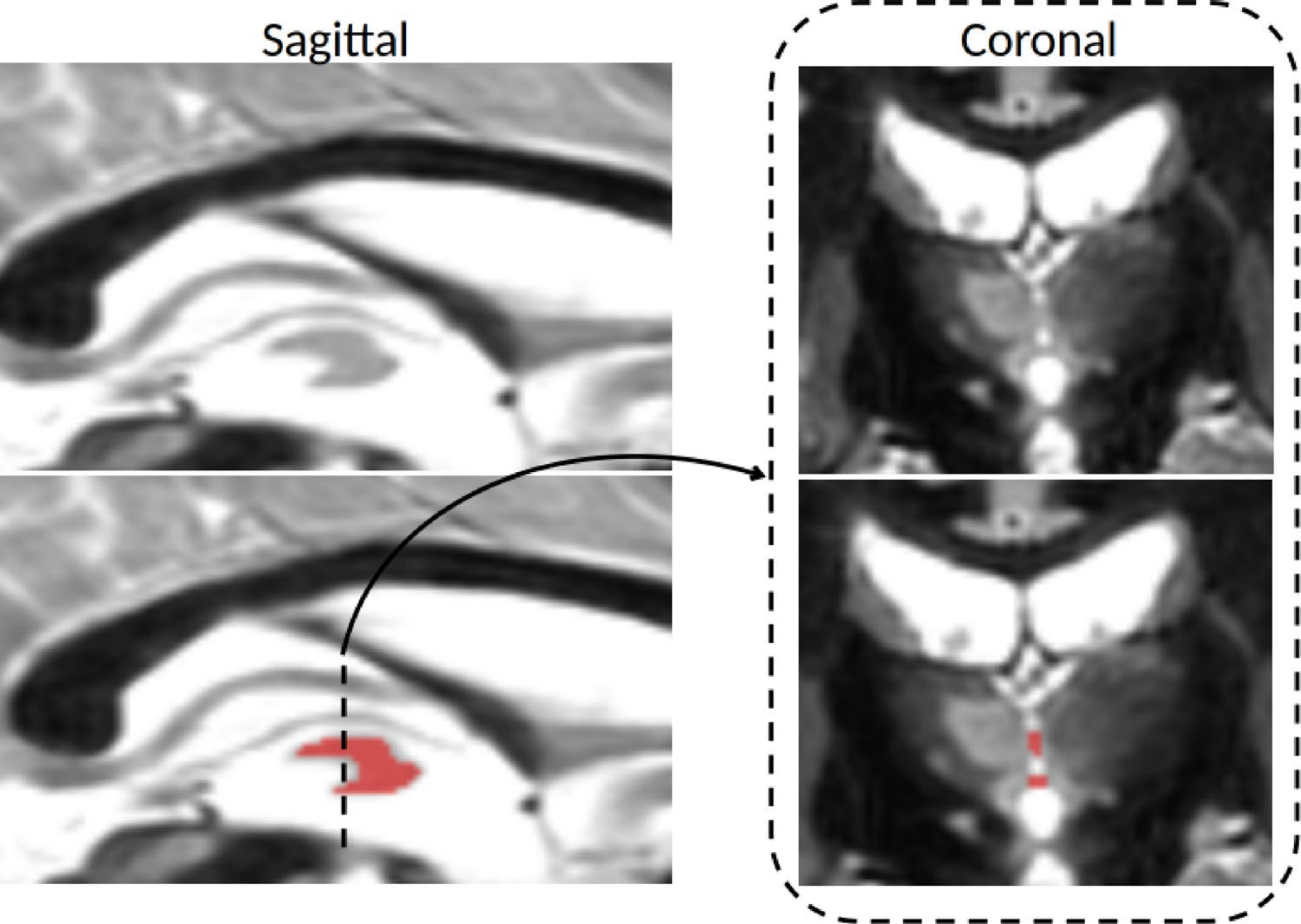
Illustration of the bilobar form of the IA on a sagittal WMn-MPRAGE slice (Planche et al. 2020; Supp. Figure 2). The dashed line on the sagittal slice indicates the corresponding coronal slice, where the two lobes appear split, potentially leading to misinterpretation as a double variant. The left top image displays the original MRI slices without segmentation, whereas the bottom image presents the corresponding slices with manual IA, segmentation performed on the sagittal slice, to enhance visualization

The *bilobar* shape, as defined by Tsutsumi and colleagues (2021), is characterized by the presence of two lobes visible in sagittal slices. Although this variant is rare and is therefore excluded from our main analyses, its identification remains important. In particular, it requires careful differentiation from the double IA variant, which presents as two distinct adhesions on axial or coronal views. In bilobar cases, the lobes may appear separated in these planes, leading to potential misclassification. Special caution is needed to avoid mistaking adjacent thalamic tissue for a second lobe in sagittal views, especially near the third ventricle, where thalamic atrophy or small size may mimic a bilobar appearance (Fig. 7).

##### A new filiform anatomical variant

**Fig. 8:**
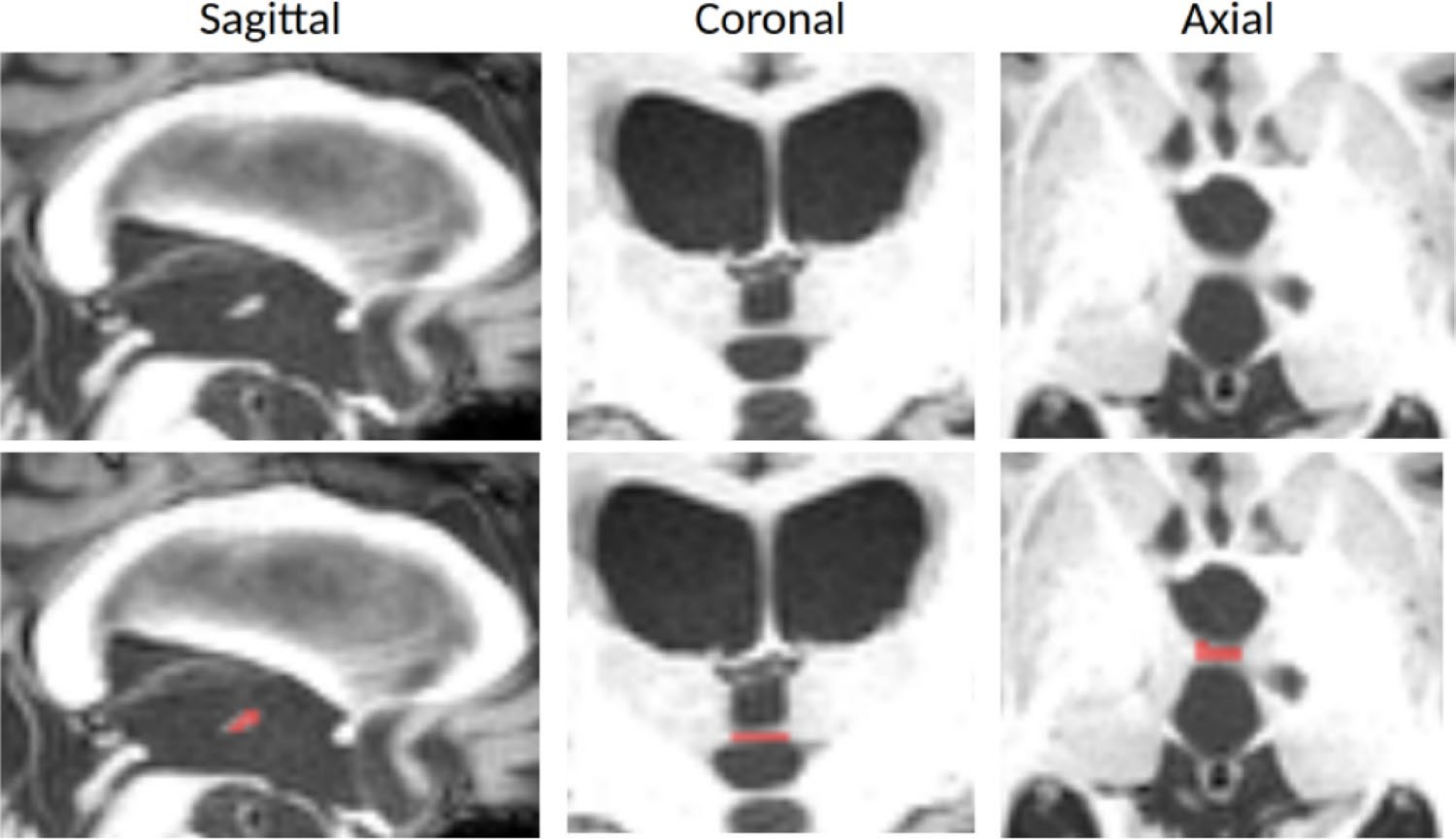
Illustration of the filiform variant of the IA on sagittal, coronal and axial T1w MRI from a right thalamic stroke patient of the Toulouse dataset (Vidal et al. 2024). The top row displays the original MRI slices without segmentation, whereas the bottom row presents the corresponding slices with manual IA segmentation realized on the sagittal slice to enhance visualization

An additional thin and elongated variant was identified and named “filiform.” This variant was defined as an IA measuring at least 4 mm in length (equivalent to 4 voxels of 1 mm) leading to at least 3 sagittal slices where the IA is clearly visible without thalamic signal, a maximum width of 2 mm on axial slices, and a maximum height of 2 mm on coronal slices. The filiform variant is particularly interesting to assess, as it may reflect IA thinning due to thalamic atrophy, ventricle enlargement, intracranial pressure (e.g. Normal Pressure Hydrocephalus) or age-related changes. In such cases, the thalami are typically further apart than in other subjects (Fig. 8). Because the rudimentary form described by Tsutsumi et al. (2021) was not observed in our data, it was not included among the assessed variants. However, this form may represent an extreme manifestation of the filiform variant, potentially corresponding to a rupture of the IA.

##### Variants interrater agreement

To ensure interrater agreement and validate the reliability of results, a kappa score can be computed on IA variants identification and must reach at least 0.70 before any consensus attempts. This threshold is based on our experience with training students and aims to reflect a score of quality. Below, it indicates excessive variability in assessment, suggesting the need for additional rater training to improve consistency and reliability.

#### Step 3: area assessment

##### Sagittal slice segmentations

The IA segmentation should be performed on sagittal slices, where it is most distinguishable from surrounding thalamic tissue, and validated on coronal and axial views. All sagittal slices that allow clear IA distinction should be segmented, with separate masks created for each slice by both raters. To ensure inter-rater segmentation consistency, a Dice index (2*intersection / union) between each rater’s segmentation masks should be computed. If the index is superior to 0.80, the overlap of raters’ masks is used to extract the area of the IA; if it is inferior to 0.80, both raters must first find an agreement on the IA boundaries. When MRI resolution is not 1 mm isotropic, the IA area (in mm²) must be adjusted by the in-plane voxel dimensions (e.g., slice width × slice height). For instance, the mean IA area in the HCP dataset which has a 0.7 × 0.7 mm resolution is calculated as: Area = Number of voxels in the IA mask × 0.49 mm². This adjustment ensures that area comparisons across datasets are anatomically accurate and not biased by voxel size differences.

##### Areas measurements & Partial volume effect (PVE)

Given the proximity of the IA to the third ventricle, the risk of PVE due to the cerebrospinal fluid (CSF) is significant. This risk depends on the rater’s subjective evaluation of voxels near the borders of the IA. To mitigate this, the use of FSL FAST (FMRIB’s Automated Segmentation Tool, Zhang et al. 2001) is recommended for modeling PVE for each voxel across different tissue classes:

- Input and Output: The input for FSL FAST is a T1w MRI scan. The tool generates three partial volume images, each containing values ranging from 0 to 1. These values represent the proportion of white matter, gray matter, and CSF present in each voxel.
- Mask Overlap: The IA mask is then overlaid onto these three partial volume images. The mean values of white matter, gray matter, and CSF within the IA mask are computed.
- PVE Correction: To accurately measure the IA area while accounting for PVE, the number of voxels is adjusted by subtracting the mean CSF proportion. This is calculated as: Adjusted Area = Number of Voxels × (1 – Mean CSF Proportion).
- Multiple Masks: If there are multiple segmentable sagittal IA masks, the areas should be averaged to account for potential spatial variations in shape.
- Biological sex differences: To account for brain size differences between sexes, IA measurements should be corrected, for example using estimated total intracranial volume (eTICV) derived via the FreeSurfer pipeline. In our study, IA areas were corrected accordingly only when analysing statistical differences between biological sexes among healthy subjects, in order to prevent introducing bias in samples where ICV and global brain size may be affected. Additionally, given the relationship between cerebrospinal fluid and IA reported in the literature (Cheng et al. 2010; Yamada et al. 2025), we suggest it is important that related results be reproduced both with and without such corrections to increase confidence in the findings.
- Additional Measurements: Length and height measurements can be obtained using the ruler tool in 3D Slicer. Furthermore, these segmentation masks can serve as seeds for diffusion imaging analyses, as described in previous studies (Damle et al. 2017).

### Statistics

To compare quantitative results between two or more groups, Mann–Whitney or Wilcoxon tests were used when data did not follow normality assumptions, while a t-test or ANOVA were used otherwise. For comparisons of qualitative variables, chi-square (χ²) tests were applied. Logistic regression analyses were used to identify predictors of the presence and absence or the IA, while an ANCOVA was preferred to study IA’s area variations. The variables considered included age, gender, and group status (patients vs. healthy controls). Statistical results are represented either using Python (Pycharm 2024.3.4) or Jasp (Version 0.19.3).

##  Results

IA segmentations and characterizations performed using SNAP-IA, are made publicly available (https://github.com/Julievidal8). Datasets are publicly available or can be provided upon request.

### Learning curves and time needed to assess the IA

An essential criterion for any protocol is the ease and speed with which new users can achieve operational proficiency. We assessed different aspects of users’ learning curves which are reported in Supplementary material. Overall, the increasing kappa scores against the expert during training for all rater pairs, along with consistently high scores between raters during assessment, demonstrate that SNAP-IA enables accurate evaluation and reduces the inherent variability in characterizing the IA.

**Fig. 9:**
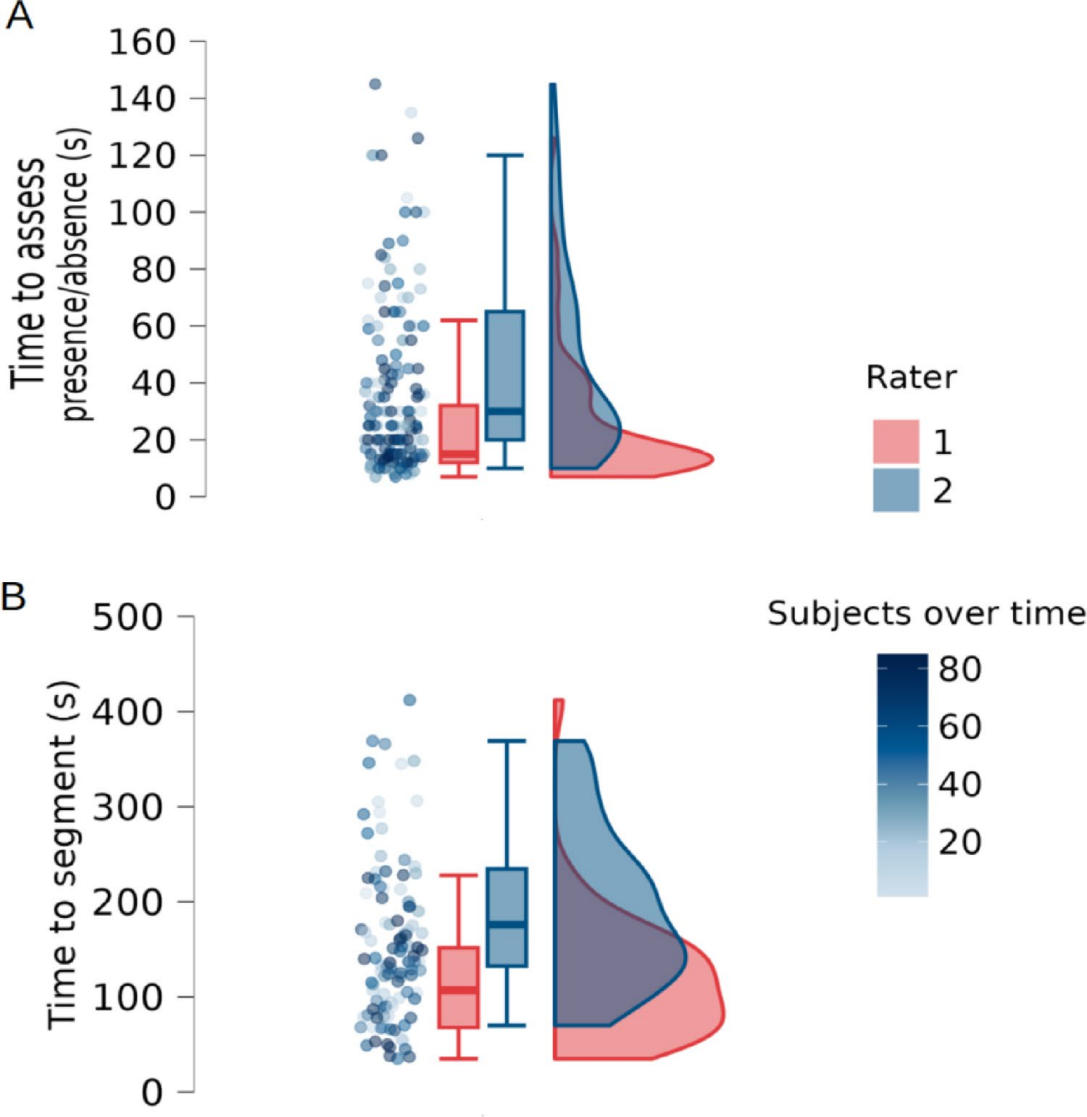
Raincloud plot (swarmplot, boxplot, and density plot) of IA characterization on all subjects of Toulouse dataset. Mean time (in seconds) required to (A) assess and (B) segment the IA by each rater. Color scales indicate the order in which each subject was assessed over time. Segmentations were performed only when the IA was present, on up to two sagittal slices in this dataset depending on its visibility

The time required to assess and segment the IA for the first pair of raters on Toulouse dataset is represented in Fig. 9 and summarized in Supp. Table 5, along with the mean Dice and Kappa scores. The average time to characterize the IA was 34 ± 23 s for healthy subjects and 36 ± 26 s for patients. Segmentation required 143 ± 61 s for healthy subjects and 139 ± 68 s for patients.

###  Validation on the general population

#### IA prevalence

Our results showed an overall IA absence rate of 22.8% in all healthy individuals among all datasets acquired at 3T (*n* = 303), an absent rate aligned with 5 prior post-mortem studies (*n* = 389) reporting an absence rate of 26.2 +- 14.9% (Wong et al. 2021). These results highlight both an internal validation as the prevalence converges across our datasets (Table 1) but also support external validation of our protocol by leveraging previous post-mortem studies results.

11 subjects were excluded due to the presence of kissing thalami that was not explained by poorer metrics during the quality check.

#### IA prevalence depending on gender

Females exhibited a significantly higher IA prevalence (84.4%) compared to males (69.8%) (females: *n* = 154; males: *n* = 149; Χ² = 9.2; *p* = 0.002), consistent with previous reports of sexual dimorphism of the IA (Borghei et al. 2021; Ceyhan et al. 2008; Davie and Baldwin 1967; Nopoulos et al. 2001; Şahin et al. 2023; Vidal et al. 2025).

To further determine which variable, age or gender, best predicted the presence or absence of the IA, a logistic regression analysis was performed including both factors as covariates (*p* = 0.007, AIC = 427, ∆Χ² = 9.9). Only gender emerged as a significant predictor, with males being approximately twice less likely to present an IA (*p* = 0.002, OR = 0.47).

**Table 1:** IA characterization among datasets

TLSE 3T	*N*	Gender (F-M)	Mean age (min-max)	% Presence	% Simple	% Broad	% Double	% Filiform
HC	45	25 − 20	48.5 (20–69)	75.6	91.2	5.8	2.9	0
Stroke	40	15–25	51 0.1 (23–75)	75.0	56.7	13.3	16.7	13.3
UCLA 3T								
HC	121	56–65	31.6 (21–50)	84.3	68.3	19.8	11.9	0
SCZ	46	12–34	36.9 (22–49)	73.9	75.8	15.2	9.1	0
BD	47	20–27	35.8 (21–50)	72.3	88.2	8.8	2.9	0
ADHD	40	20–20	32.6 (21–50)	77.4	87.1	6.5	3.2	3.2
HCP YA 3T								
HC	137	73 − 64	29.0 (22–35)	71.5	74.5	25.5	0	0
9.4T								
HC	78	28–50	30.4 (20–53)	65.4	86.5	13.5	0	0
TOTAL HC 3T	303	154 − 149	32.9 (20–69)	77.2	74.3	20.2	5.6	0

##### Anatomical variants

For IA variant assessment, one case in the UCLA dataset was excluded due to inter-rater discordance or low confidence from kissing thalami. Among healthy 3T subjects where the IA was present, the simple and broad variants predominated (94.5%; Table 1), a finding consistent with prior literature (Tsutsumi et al. 2021).

##### IA areas

The dimensions of the IA have been previously reported to vary widely, from 3 to 22 mm in length and 1.5 to 12.5 mm in height (Borghei et al. 2020; Patra et al. 2022; Pavlović et al. 2020; Takahashi et al. 2008a, b). However, comparisons across studies are challenged by the lack of standardized criteria for defining measurement axes. The present protocol addresses these concerns by emphasizing area estimation on sagittal slices and correcting for PVE, offering a more objective and reproducible metric.

The mean dice reflecting the agreement of IA’s segmentation between the two raters were on average 0.92, ensuring reliable area analyses. Mean area corrected for the PVE demonstrated an averaged area across all variants and healthy subjects acquired at 3T of 22.7 *±* 11 mm^2^ (Table 2). Female subjects had significantly larger IA areas than males (24.5 ± 10.7 vs. 20.5 ± 11.8 mm²; U = 7995, *p* = 0.003, *n* = 230). This difference was further increased after correcting areas for eTICV (U = 9215, *p* < 0.001), reinforcing established sex-based anatomical distinctions (Pavlovic et al., 2020; Tsutsumi et al. 2021). Notably, a general linear model confirmed that there was no correlation between eTICV and PVE-corrected IA area after accounting for age and sex (*p* = 0.913).

**Table 2:** IA’s mean (SD) area adjusted for PVE across anatomical variants in mm2. N are after exclusions

TLSE 3T	*N*	Mean dice (min-max)	Mean area	Simple	Broad	Double	Filiform
HC	45	0.92 (0.83-1)	17.2 (10)	16.4 (9)	33.8 (9)	9	NA
Stroke	40	0.93 (0.84-1)	16.4 (11)	14.7 (8)	37.8 (7)	14.3 (3)	4.4 (2)
UCLA 3T							
HC	121	0.94 (0.80-1)	24.5 (12)	19.9 (9)	36.0 (8)	33.2 (18)	NA
SCZ	46	0.94 (0.85-1)	20.5 (10)	18.4 (10)	30.2 (6)	21.0 (5)	NA
BD	47	0.93 (0.80-1)	20.7 (9)	19.9 (9)	29.3 (3)	17.9	NA
ADHD	40	0.93 (0.80-1)	22.0 (14)	19.2 (7)	58.5 (23)	42.1	3.3
HCP YA 3T							
HC	137	0.88 (0.80-1)	23.0 (10)	18.7 (7)	35.6 (9)	NA	NA
9.4T							
HC	78	0.92 (0.81-1)	19.5 (11)	16.4 (9)	39.4 (6)	NA	NA
TOTAL HC 3T	303	0.92 (0.80 -1)	22.7 (11)	18.8 (8)	35.7 (8)	31.3 (19)	NA

**Fig. 10:**
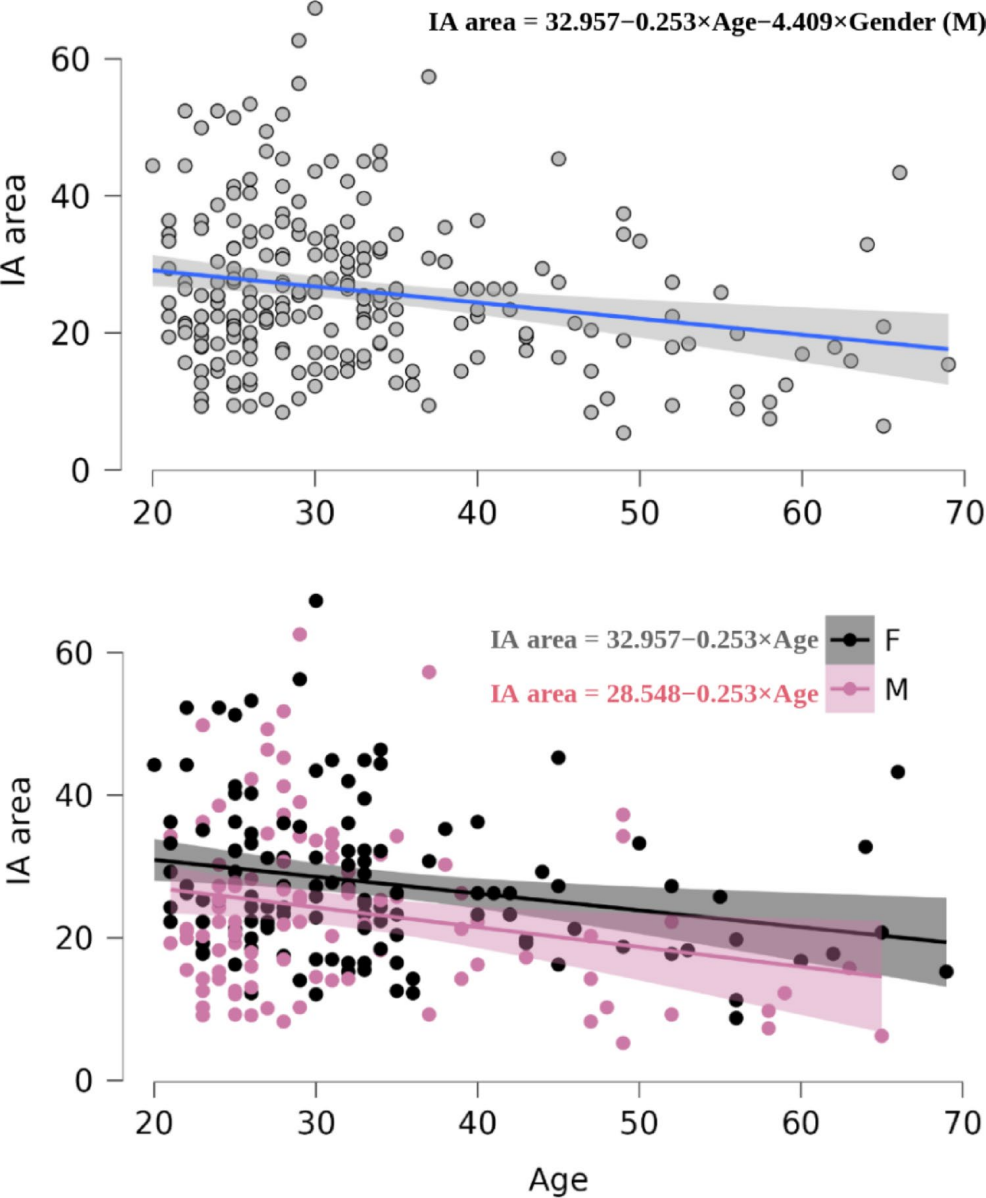
A- Dotplots of IA area (mm^2^) by age along with means and 95% confidence intervals independently of genders and B- depending on genders. F: females; M: males

An ANCOVA was conducted to assess the predictive value of age and gender on IA area. Both factors were significant predictors (gender: *p* = 0.003, ω² = 0.033; age: *p* < 0.001, ω² = 0.050; Fig. 10A–B). On average, the IA area decreased by 0.25 mm² for each additional year of age (t = − 3.68), with females having a larger IA area than males, independently of age. The effect of age on IA area is consistent with previous, though sometimes debated, reports of age-related decline in IA area (Sen et al. 2005; Trzesniak et al. 2011, 2016; Tsutsumi et al. 2021).

Overall, among all healthy subjects across all 3T datasets, the mean IA area of simple IA differed, as expected by definition, significantly from broad and double variants (ANOVA, t-tests with Bonferroni correction; *p* < 0.001, Cohen’s d = 1.9 and 1.4). This supports our confidence in the protocol’s ability to reliably distinguish these variants but also demonstrates its relevance.

###  Clinical validation

#### Thalamic stroke patients

This dataset included 40 patients with isolated ischemic thalamic lesions at the chronic stage and 45 controls (Danet et al. 2015; 2017; Vidal et al. 2024). Among the patients, the lesion extended into the IA in 12 cases. SNAP-IA was able to both (1) correctly identify the presence of the IA and (2) determine whether the lesion extended into it, even in complex cases involving a damaged simple IA (Supp Fig. 2) or a partially affected double IA variant (Supp Fig. 3). This confirms the method’s generalizability in the presence of lesions.

#### Neurodevelopmental and neuropsychiatric disorders

Earlier studies have suggested an association between IA absence and neurodevelopmental or neuropsychiatric disorders (Andreasen et al. 1994; Shenton et al. 2001; Trzesniak et al. 2011, 2016; Takahashi et al. 2009). We sought to replicate these findings using SNAP-IA.

The UCLA dataset (Table 1, Gorgolewski et al. 2017) included 125 healthy individuals and 140 patients with schizophrenia (*n* = 50), bipolar disorder (*n* = 49), or attention deficit hyperactivity disorders (*n* = 41) combined to increase statistical power. There were no biological sex differences between groups but healthy subjects were significantly younger (t-test, *p* = 0.002, cohen’s d= -0.4, mean age healthy subjects = 31.5 ± 8.7 vs. patients = 35.2 ± 9.5).

**Fig. 11:**
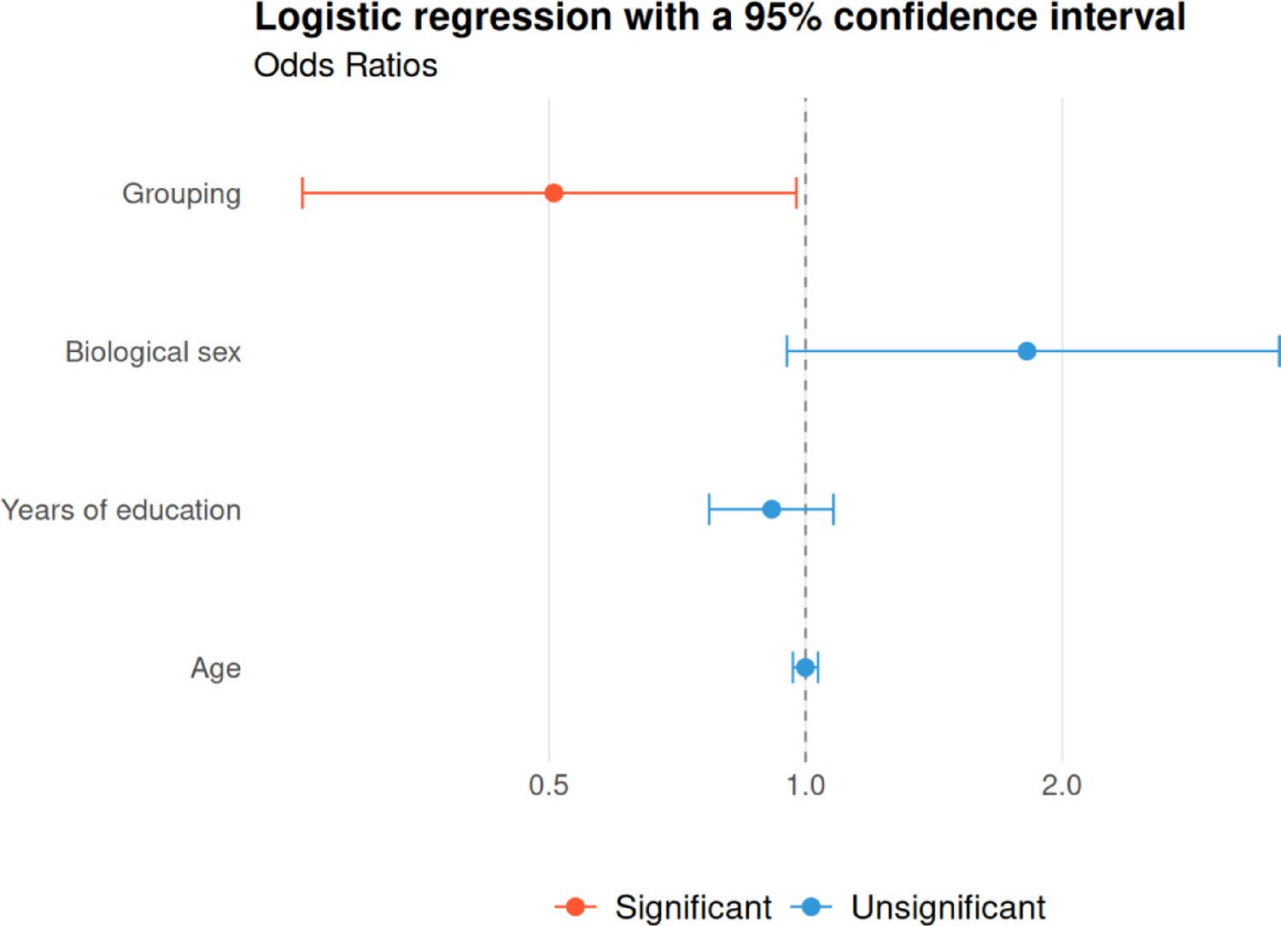
Odds Ratio with 95% confidence interval of the logistic regression predicting the IA presence depending on the group factor (neurodevelopmental and neuropsychiatric disorders patients or healthy subjects), biological sex, years of education, and age

Patients showed a significantly lower IA prevalence (74%) than healthy subjects (84%) (*p* = 0.05, Χ² = 3.7), and a significantly smaller mean corrected IA area (21.0 ± 10.9 mm² vs. 24.4 ± 12.2 mm²; *p* = 0.045, Cohen’s d = 0.29). Logistic regression including age, education, gender, and group (patients vs. healthy) revealed that individuals with brain disorders were about half as likely to present an IA (OR = 0.51, *p* = 0.045), with no other significant predictors. A trend was observed for gender, with males nearly twice as likely to lack an IA (OR = 1.8, *p* = 0.076). These results are consistent with prior literature and support the applicability of SNAP-IA in these patient groups (Fig. 11).

###  Validation at different field strength and resolution

To evaluate whether SNAP-IA could be extended to higher field strengths and whether results would align with conventional 3T findings, we compared a 9.4T submillimeter dataset (*n* = 78, age 20–53, 0.6–0.8 mm^3^ resolution) with a 3T dataset (*n* = 166 after exclusions, 1 mm³ resolution) composed of healthy subjects from the UCLA and Toulouse cohorts.

The groups were comparable in gender distribution (χ²=3.6, *p* = 0.059), but the 9.4T cohort was significantly younger (30.4 ± 7.1 vs. 36.2 ± 12.8 years; U = 7,723, *p* = 0.015 ; Table 1). IA absence was significantly higher at 9.4T (34.6%) than at 3T (18.1%) after adjusting for age (logistic regression, *p* = 0.002). Variant distribution did not differ significantly between field strengths (χ²=5.9, *p* = 0.053), despite only simple (86%) and broad (14%) variants identified at 9.4T.

**Fig. 12:**
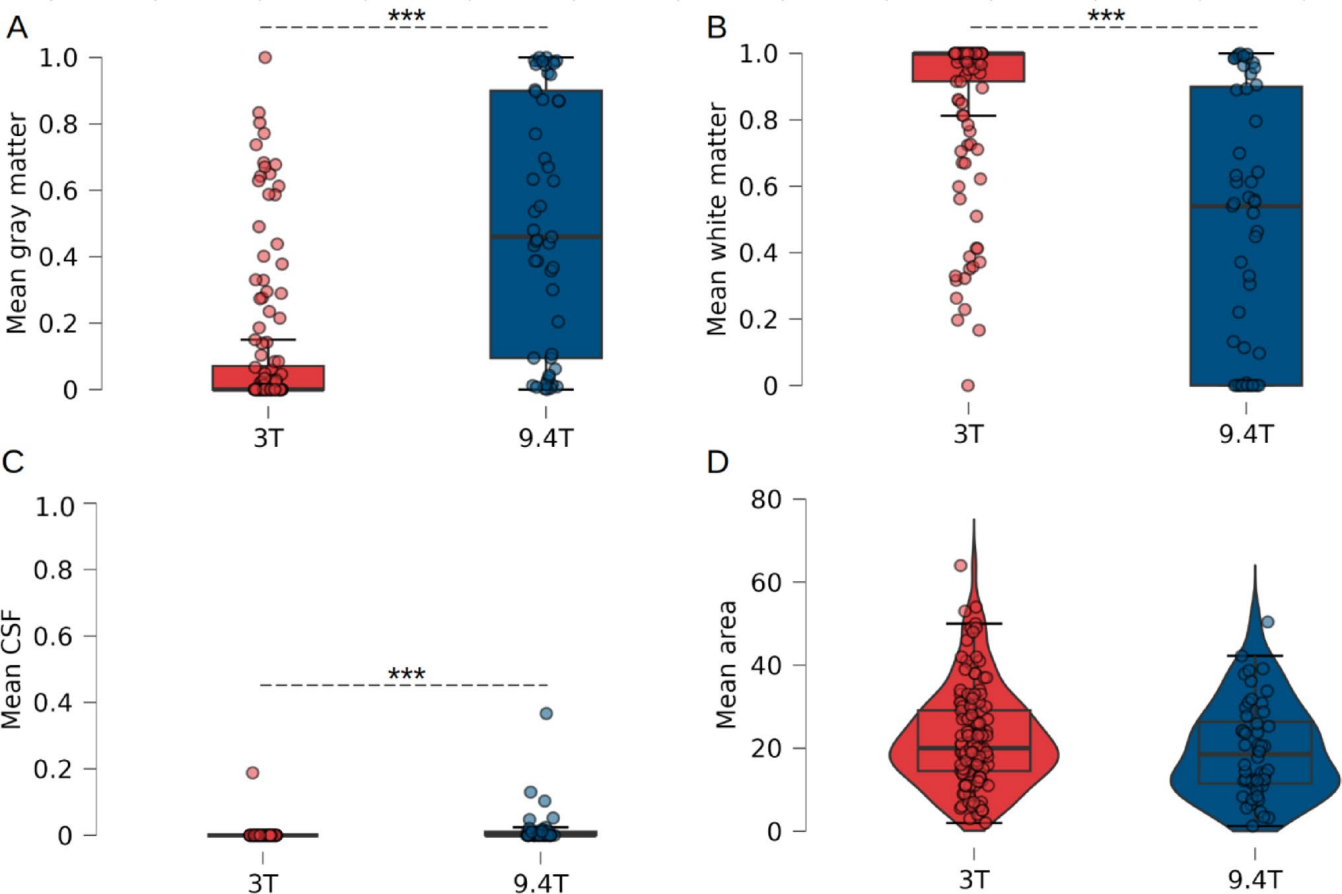
IA composition and area. A- Mean rate of gray matter, B- Mean rate of white matter C- Mean rate of CSF in the IA by resolution (3T vs. 9.4T). Those analysis leverages on a FAST FSL segmentation of each voxel into the three classes (CSF, GM, WM). D- Mean area of the IA, corrected for the PVE. ***, *p* < 0.001, Mann-Whitney or Wilcoxon test

After PVE correction, IA masks at 9.4T showed significantly higher gray matter and CSF content and lower white matter content compared to 3T (Wilcoxon, *p* < 0.001; Fig. 12A–C). However, mean IA area did not differ significantly between field strengths (Mann-Whitney U, U = 3922, *p* = 0.132; Fig. 12D; Table 2).

To ensure that the higher absence rate was not due to the submillimeter resolution of the 9.4T dataset, we compared it with the HCP dataset acquired at 3T with a comparable resolution of 0.7 mm³ (*n* = 137, age 22–35). Age was comparable between datasets (Mann–Whitney U test, *p* = 0.662), although the HCP dataset contained a higher proportion of females (53% vs. 47%; χ² = 6.0, *p* = 0.014). No significant differences were observed in IA absence rate (χ² = 3.6, *p* = 0.347), IA variant distribution (χ² = 2.9, *p* = 0.086), or mean IA area (Mann–Whitney U = 2052, *p* = 0.05).

## Discussion

This study introduces SNAP-IA (Standardized Neuroanatomical Assessment Protocol), an updated and rigorously validated version of our earlier protocol (Vidal et al. 2024) for the reliable assessment of the IA on anatomical MRI. SNAP-IA was applied to 565 individuals, including healthy cohorts, thalamic stroke patients, and patients with neurodevelopmental or neuropsychiatric disorders. By integrating standardized criteria for IA presence/absence, morphological classification, and area measurement with PVE correction, SNAP-IA addresses long-standing methodological inconsistencies in IA research.

### Key findings and validation

The protocol’s ease of application and teaching has been demonstrated through successful use in over 1,000 (un)published cases by independent research groups, confirming its reliability and generalizability in large-scale, multi-domain studies.

Our findings converge with post-mortem evidence, confirming an IA absence rate of ~ 23% in healthy individuals, with simple and broad variants accounting for the vast majority of cases (Wong et al. 2021; Tsutsumi et al. 2021). In line with prior literature, IA prevalence was significantly lower in males and in patients with neurodevelopmental or neuropsychiatric disorders, the latter showing a twofold reduction in IA presence and significantly smaller IA size (Borghei et al. 2021; Şahin et al. 2023; Trzesniak et al. 2011; Takahashi et al. 2009). We observed an age-related decline in IA area of − 0.25 mm²/year (1.1% annual loss), a rate approximately double the typical thalamic volume loss seen in normal aging (Schippling et al. 2017). Females showed both higher IA prevalence and larger area (Borghei et al. 2021).

Across datasets and field strengths, segmentation consistency remained high (mean Dice ≈ 0.92). Submillimeter 9.4T MRI yielded a higher IA absence rate (34.6%) compared to 3T (18.1%). However, our comparison with the HCP 3T dataset acquired at comparable submillimeter resolution revealed no significant differences. This suggests that the higher absence rate observed at 9.4T is not attributable to improved resolution per se, but rather to the inherent contrast enhancement and sharper delineation of structural boundaries afforded by high field imaging. Such contrast benefits are particularly valuable for resolving ambiguous cases such as kissing thalami. These findings may also be partly influenced by the younger age and smaller cohort size of the 9.4T sample, warranting further investigation. PVE-corrected IA area remained stable, underscoring the protocol’s robustness. Notably, post-mortem studies, the best available ground truth, report an intermediate absence rate of ~ 26% (Wong et al. 2021), though formalin fixation may artifactually reduce IA prevalence (Trzesniak et al. 2011).

### Training requirements and assessment challenges

Training proved essential for reliable IA assessment. The target inter-rater Kappa score of 0.7 for variant identification was reached only after ~ 115 assessments and remained stable thereafter, highlighting both the protocol’s robustness and the need for rigorous rater calibration.

Challenges were most pronounced when the thalami are closely positioned, leading to kissing thalami or stronger PVE, producing signals in sagittal views that can easily mimic the appearance of an IA, increasing subjectivity in determining IA presence and classifying variants. These anatomical configurations, combined with variability in MRI acquisition parameters and assessment methodologies, contribute to the wide range of IA rates reported in the literature.

### Variant classification vs. quantitative area measurement

While variant classification remains informative, especially in clinical contexts like thalamic stroke, where only one IA may be preserved in double variant cases, it is inherently subjective with high influence of thalamic proximity. Moreover, the high intra- and inter-individual variability in thalamic shape and size further complicates visual classification, emphasizing the need for sagittal classification and segmentation for reliable IA and variant identification. In rare cases where the IA lies diagonally on sagittal slices, variant classification may be inaccurate despite similar area measurements, reinforcing the subjectivity of categorical classification. In contrast, quantitative area measurement, especially with PVE correction, provides a more robust, objective, and generalizable metric for IA characterization.

### IA composition

IA masks at 9.4T contained significantly higher proportions of CSF compared to 3T, independent of rater or image quality differences. These findings emphasize the necessity of PVE correction, particularly when comparing across field strengths. Indeed, after PVE correction, mean IA area did not differ significantly between 3T and 9.4T.

Our tissue composition analyses align with growing evidence that the IA contains both white and gray matter. Diffusion imaging and tractography studies suggest it harbors axonal fibers and contributes to interhemispheric connectivity (Şahin et al. 2023; Borghei et al. 2021; Vidal et al. 2025), while histological work has identified neuronal elements within the IA (Rabl 1958; Malobabić et al. 1987; van Heerden et al. 2025). Although MRI lacks the resolution of histology, these convergent findings underscore the IA’s potential structural and functional relevance.

### Broader implications and future directions

SNAP-IA enables standardized, cross-study comparisons, paving the way for more reliable exploration of IA morphology across the lifespan, in various pathologies, and in relation to cognition. Key future directions include:

- Cognitive and clinical correlates: While previous studies suggest links between IA absence and neuropsychological impairments (e.g., attention, memory), the SNAP-IA framework provides a means to revisit these associations with better anatomical reliability.
- Structural and functional connectivity: Using IA masks as seeds in diffusion MRI and resting-state fMRI to investigate structural and functional connectivity, avoiding bias from using adjacent structures (e.g., habenula). Diffusion imaging could allow to study not only IA’s area but also its volume, which is limited in anatomical MRI due to insufficient contrast where the IA traverses the thalami. Then, stratifying connectivity analyses by IA presence and size may yield insights into its role in brain network organization.
- The filiform variant: Longitudinal studies should assess whether this newly described variant reflects age-related (i.e. − 0.25 mm²/year) or pathological thinning related to thalamic atrophy, ventricular enlargement, or neurodegeneration. Confirming the absence of connectivity in such cases, as suggested by Kochanski et al. (2018), would be valuable.
- Extension to Other Midline Structures: Applying the SNAP framework to other midline commissures (e.g., interhypothalamic adhesion, mammillary body adhesion) could provide integrated insights into interhemispheric connectivity and compensatory mechanisms.
- Automation and Scalability: The training demands underscore the potential of machine learning. Automated segmentation models trained on SNAP-IA ground-truth masks could streamline IA assessment and facilitate large-scale studies.

## Conclusion

This study introduces SNAP-IA, a standardized protocol for the anatomical MRI assessment of the interthalamic adhesion (IA). Applied to 565 participants across multiple datasets and pathologies, the protocol demonstrated strong inter-rater reliability and easy applicability to all cases.

## Supplementary Information

Below is the link to the electronic supplementary material.

Supplementary Material 1

## Data Availability

A detailed video protocol outlining the IA characterization process is publicly available ( [https://github.com/Julievidal8/SNAP-IA-protocol](https:/github.com/Julievidal8/SNAP-IA-protocol) ). Datasets, including IA segmentations and characterizations performed using SNAP-IA, are made publicly available or provided upon request ( [https://github.com/Julievidal8](https:/github.com/Julievidal8) ).
