# Supplementary material for: Standardized methodology for assessing the presence, variants and area of the interthalamic adhesion using anatomical MRI (SNAP-IA): multicentric validation on 565 healthy individuals and multiple neurological disorders
Source: Brain Struct Funct. 2026 Mar 23;231(3):42. doi: 10.1007/s00429-026-03097-6 (PMC13009120; doi:10.1007/s00429-026-03097-6)

Standardized methodology for assessing the presence, variants and area of the Interthalamic Adhesion using anatomical MRI (SNAP-IA): multicentric validation on 565 healthy individuals and multiple neurological disorders

**SUPPLEMENTARY MATERIAL**


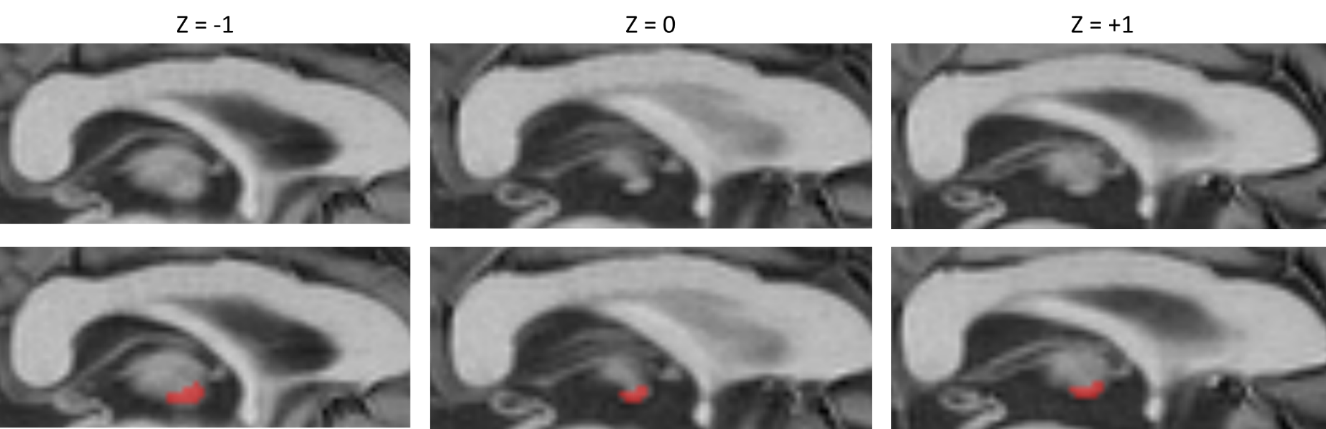


**Supplementary Figure 1:** Sagittal MRI slices from a single subject illustrating thalamic size variation through three consecutive slices, the mid-thalamic slice (Z = 0), the preceding slice (Z = –1), and the following slice (Z = +1). The IA maintains a relatively consistent size and shape across the images. The top row shows the original MRI images, while the bottom row presents the corresponding images with manual IA segmentation for enhanced visualization.

**Learning curves**

**UCLA cohort**

The first pair of raters (students 1.1 and 1.2) were trained to assess the IA using Toulouse dataset, as well as 30 subjects from the UCLA database, leading to 115 assesments. All subjects were also annotated by J.P.V., whose annotations served as the ground truth for validation purposes during training (kappa vs expert in table 1) while kappa score are computed between both raters during assessment. The progression of the Kappa scores, both during the training phase and subsequent assessments, shows a constant increase, indicating effective learning and improved consistency between the two raters (see Supp. Tab. 1 & 2).

This first pair of students was instrumental in defining both the number of assessments required for adequate training and the criteria to evaluate acquired expertise. Initially, the training was intended to rely solely on Toulouse dataset. However, to ensure the generalizability of the training process across different imaging conditions, we included a subset of the UCLA dataset. This addition led to the computation of two Kappa scores based on 15 UCLA subjects, making the 0.7 threshold more challenging to reach and explaining the slightly lower scores in that portion of the training. Indeed, with such a small sample size, each disagreement between raters has a disproportionately large impact on the Kappa value, making it harder to achieve high agreement scores. To improve consistency, we subsequently decided to compute Kappa scores every 50 subjects. By the end of the training phase (after 115 assessments), both raters had reached the target Kappa score of approximately 0.7 for IA variant classification (Supp. Tab. 1), ensuring reliable performance in the UCLA evaluation phase, where this threshold was consistently maintained (Supp. Tab. 2).

***Supp. Tab. 1*** *: Training progression about the IA characterization (presence and variant) of the pair of raters 1 reflected by their kappa’s score against an expert (J.P.V.) on different T1w MRI datasets. A Kappa score above 0.70 indicates a high level of agreement with the expert. Scores below 0.70 are underlined and colored in red, signaling the continued need for training to reduce variability and enhance reliability in IA characterization.*

| **TRAINING** | **Datasets** | **Rater 1.1**  **IA presence** | **Rater 1.2**  **IA presence** | **Rater 1.1**  **IA variant** | **Rater 1.2**  **IA variant** |
| --- | --- | --- | --- | --- | --- |
| **Kappa vs expert** | *Toulouse HS (N=45)* | 0.25 | 0.31 | 0.15 | 0.25 |
|  | *Toulouse*  *Patients (N=40)* | 1 | 1 | 0.69 | 0.7 |
|  | *UCLA 1-15* | 1 | 0.87 | 0.56 | 0.56 |
|  | *UCLA 15-30* | 1 | 1 | 0.9 | 0.67 |

***Supp. Tab. 2*** *: Kappa’s score progression of raters’ pair 1 during the IA assessment on the UCLA T1w MRI dataset. A Kappa score above 0.70 indicates a high level of agreement between both raters.*

| **ASSESSMENT** | **UCLA subjects** | **IA presence** | **IA variant** | **IA presence after consensus** | **IA variant after consensus** |
| --- | --- | --- | --- | --- | --- |
| **Kappa rater 1.1 vs 1.2** | *30-50* | 0.77 | 0.78 | 1 | 1 |
|  | *50-100* | 1 | 0.78 | 1 | 1 |
|  | *100-150* | 0.89 | 0.72 | 0.94 | 0.96 |
|  | *150-200* | 0.92 | 0.85 | 0.95 | 0.92 |
|  | *200-250* | 0.92 | 0.92 | 0.78 | 0.89 |
|  | *250-275* | 1 | 1 | 1 | 1 |

**HCP YA & Dallas cohorts**

The second pair of raters (students 2.1 and 2.2) were trained to assess the IA using Toulouse dataset, as well as 50 subjects from the publicly available Dallas database and 37 subjects from the HCP YA database, leading to an overall training on 172 subects. All subjects were also annotated by J.P.V., whose annotations served as the ground truth for validation purposes during training (kappa vs expert in Supp. Tab. 1) while kappa score are computed bewteen both raters during assessment. The progression of the Kappa scores, both during the training phase and subsequent assessments of both Dallas and HCP datasets, shows a consistent increase, indicating effective learning and improved consistency between the two raters (Supp. Tab. 3 & 4). The target Kappa score of approximately 0.7 for IA variant characterization during training was quickly reached for the first rater and almost reached for the second rater after 172 assessments (Supp. Tab. 3), ensuring reliable performance during the HCP evaluation (Supp. Tab. 4), where the threshold was consistently maintained.

***Supp. Tab. 3*** *: Training progression on the IA characterization (presence and variant) of the rater pair 2 reflected by their kappa’s score against an expert on different T1w MRI datasets. A Kappa score above 0.70 indicates a high level of agreement with the expert. Scores below 0.70 are underlined and colored in red, signaling the continued need for training to reduce variability and enhance reliability in IA characterization.*

| **TRAINING** | **Datasets** | **Rater 2.1**  **IA presence** | **Rater 2.2**  **IA presence** | **Rater 2.1**  **IA variant** | **Rater 2.2**  **IA variant** |
| --- | --- | --- | --- | --- | --- |
| **Kappa vs expert** | Toulouse HS (N=45) | 0.72 | 1 | 0.64 | 0.42 |
|  | Toulouse  Patients (N=40) | 1 | 1 | 0.73 | 0.66 |
|  | DALLAS 0-50 | 1 | 0.78 | 0.85 | 0.62 |
|  | HCP 0-37 | 1 | 0.85 | 0.78 | 0.62 |

***Supp. Tab. 4*** *: Kappa’s score progression of rater pair 2 during the IA assessment on the HCP T1w MRI dataset (Van Essen et al., 2013). A Kappa score above 0.70 indicates a high level of agreement between both raters.*

| **ASSESSMENT** | **HCP subjects** | **IA presence** | **IA variant** | **IA presence after consensus** | **IA variant after consensus** |
| --- | --- | --- | --- | --- | --- |
| **Kappa rater 2.1 vs 2.2** | 0-50 | 1 | 0.85 | 1 | 1 |
|  | 50-100 | 1 | 0.96 | 1 | 1 |

***Supp. Tab. 5 :*** *IA methods information on Toulouse dataset. Mean time in seconds (s). Segmentations were realized by two raters when the IA is present, on one and up to two sagittal slices in this dataset depending on how clearly the IA was visible. Kappa were computed before attempts to reach a consensus.*

|  | ***N*** | ***Mean time (SD)*** | ***Mean time to segment (SD)*** | ***Mean dice***  ***(min-max)*** | ***Kappa IA presence***  ***/ variants*** |
| --- | --- | --- | --- | --- | --- |
| HC | 45 | 34 (23) | 143 (61) | 0.92 (0.83-1) | 1 / 0.92 |
| Patients | 40 | 36 (26) | 139 (68) | 0.93 (0.84-1) | 1 / 0.93 |

**Stroke (Toulouse cohort)**

This dataset was initially characterized by K.R. and J.P.V. (Vidal et al., 2024) and was subsequently re-evaluated using SNAP-IA by raters 1.1 and 1.2 following their training. Since IA are segmented on a single sagittal slice, the lesion does not overlap with the segmented slice in the double variant but can still be visually identified as reaching the IA.

**Supplementary Figure 2:** Illustration of a damaged IA using Toulouse cohort (Vidal et al., 2024). A bilateral thalamic lesion and the IA were manually segmented by two raters. The lesion is highlighted in red, while the IA is in blue. The IA was segmented on a single sagittal MRI slice. The overlap between the lesion and the IA, representing the IA's lesion, appears in pink. The blue cross indicates the cursor positioned on the same voxel across different MRI views.


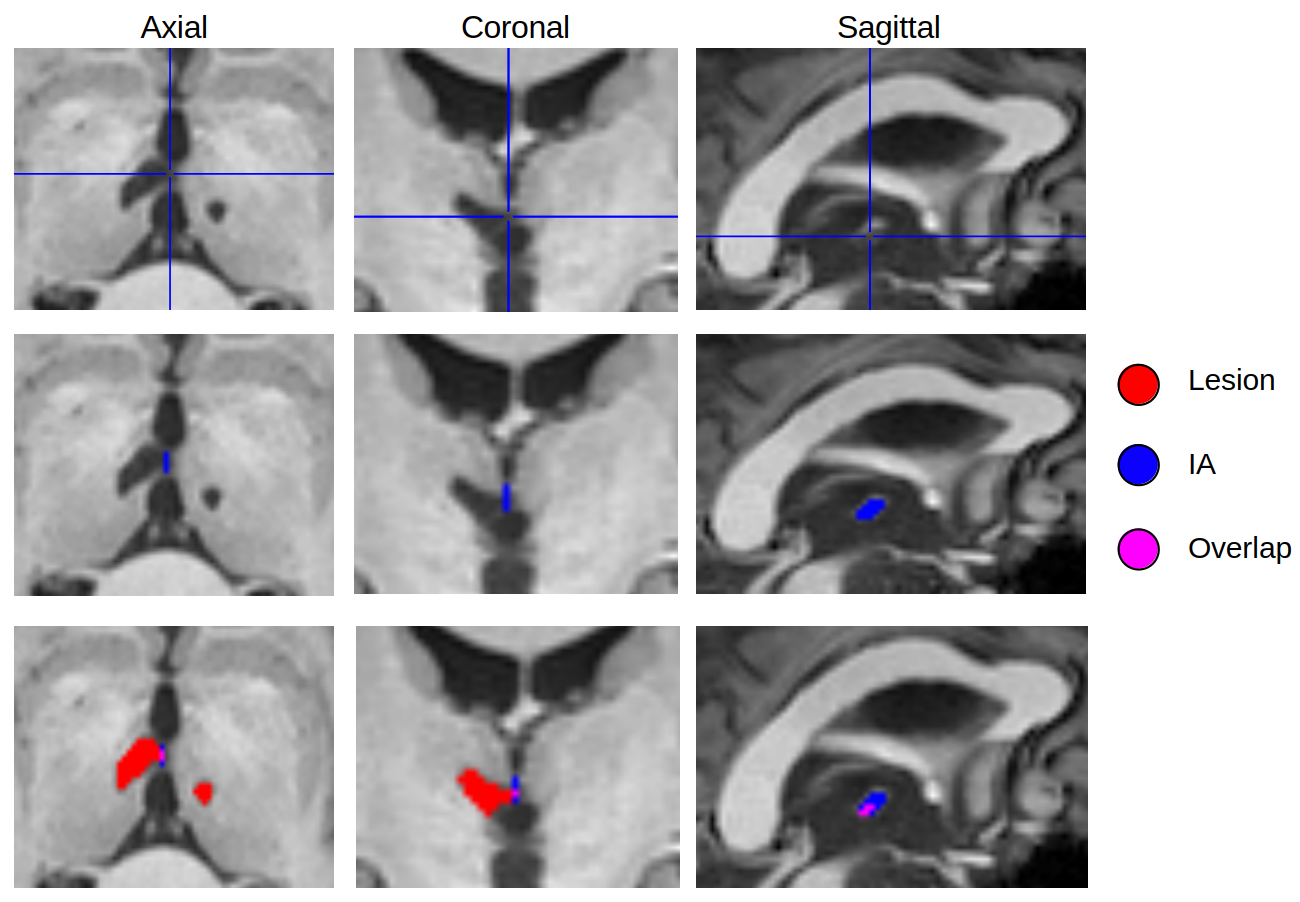


**Supplementary Figure 3**: Double IA variant with only one IA being damaged from Toulouse cohort MRI dataset (Vidal et al., 2024). The thalamic lesion and the IA were manually segmented by two raters. The lesion is highlighted in red, while the IA, segmented in a single sagittal slice, is in blue. As the IA are segmented in a single Sagittal slice, there are no overlaps with the lesion in this representation but the damaged IA is the bottom one.


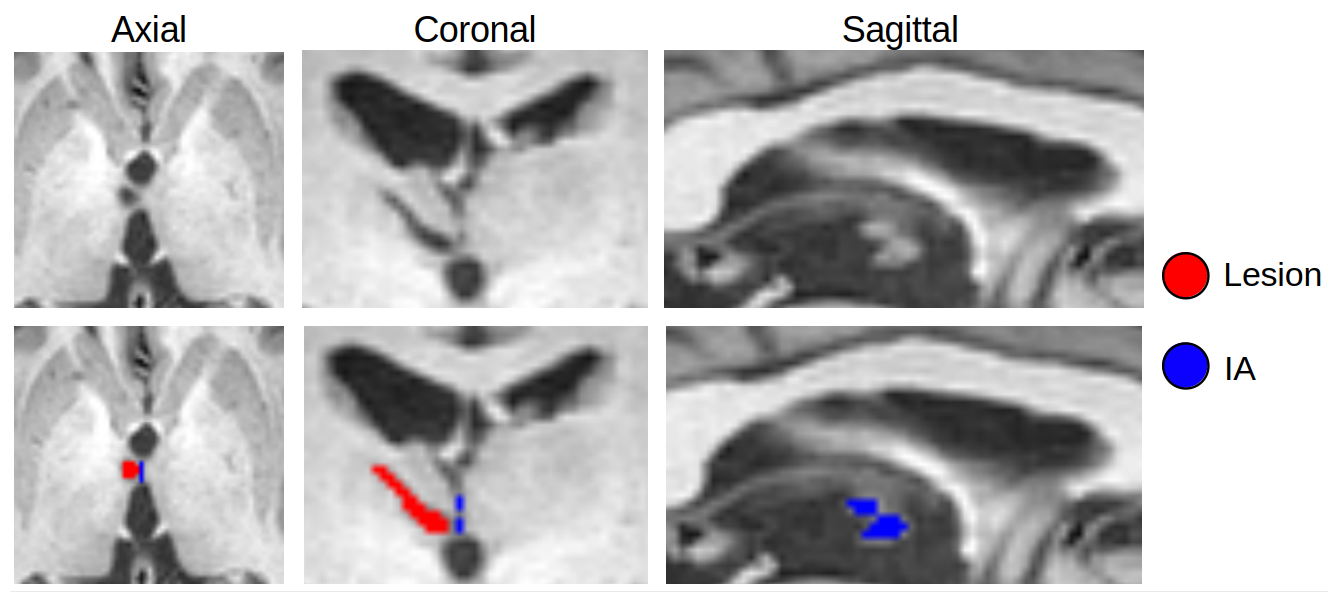

Supplement: Supplementary file 1 — Supplementary Material 1 [file 429_2026_3097_MOESM1_ESM.docx]
